# Spheroid-Induced Epithelial-Mesenchymal Transition Provokes Global Alterations of Breast Cancer Lipidome: A Multi-Layered Omics Analysis

**DOI:** 10.3389/fonc.2019.00145

**Published:** 2019-03-21

**Authors:** Yun Pyo Kang, Jung-Ho Yoon, Nguyen Phuoc Long, Gi-Bang Koo, Hyun-Jin Noh, Seung-Jae Oh, Sae Bom Lee, Hyung Min Kim, Ji Yeon Hong, Won Jun Lee, Seul Ji Lee, Soon-Sun Hong, Sung Won Kwon, You-Sun Kim

**Affiliations:** ^1^College of Pharmacy, Seoul National University, Seoul, South Korea; ^2^Department of Biochemistry, Ajou University School of Medicine, Suwon, South Korea; ^3^Department of Biomedical Sciences, Graduate School, Ajou University, Suwon, South Korea; ^4^Department of Biomedical Sciences, College of Medicine, Inha University, Incheon, South Korea; ^5^Research Institute of Pharmaceutical Sciences, Seoul National University, Seoul, South Korea

**Keywords:** breast cancer, epithelial-mesenchymal transition, lipidomics, transcriptomics, fatty acid metabolism, ELOVL2

## Abstract

Metabolic rewiring has been recognized as an important feature to the progression of cancer. However, the essential components and functions of lipid metabolic networks in breast cancer progression are not fully understood. In this study, we investigated the roles of altered lipid metabolism in the malignant phenotype of breast cancer. Using a spheroid-induced epithelial-mesenchymal transition (EMT) model, we conducted multi-layered lipidomic and transcriptomic analysis to comprehensively describe the rewiring of the breast cancer lipidome during the malignant transformation. A tremendous homeostatic disturbance of various complex lipid species including ceramide, sphingomyelin, ether-linked phosphatidylcholines, and ether-linked phosphatidylethanolamine was found in the mesenchymal state of cancer cells. Noticeably, polyunsaturated fatty acids composition in spheroid cells was significantly decreased, accordingly with the gene expression patterns observed in the transcriptomic analysis of associated regulators. For instance, the up-regulation of *SCD, ACOX3*, and *FADS1* and the down-regulation of *PTPLB, PECR*, and *ELOVL2* were found among other lipid metabolic regulators. Significantly, the ratio of C22:6n3 (docosahexaenoic acid, DHA) to C22:5n3 was dramatically reduced in spheroid cells analogously to the down-regulation of *ELOVL2*. Following mechanistic study confirmed the up-regulation of *SCD* and down-regulation of *PTPLB, PECR, ELOVL2*, and *ELOVL3* in the spheroid cells. Furthermore, the depletion of *ELOVL2* induced metastatic characteristics in breast cancer cells via the SREBPs axis. A subsequent large-scale analysis using 51 breast cancer cell lines demonstrated the reduced expression of *ELOVL2* in basal-like phenotypes. Breast cancer patients with low *ELOVL2* expression exhibited poor prognoses (HR = 0.76, CI = 0.67–0.86). Collectively, *ELOVL2* expression is associated with the malignant phenotypes and appear to be a novel prognostic biomarker in breast cancer. In conclusion, the present study demonstrates that there is a global alteration of the lipid composition during EMT and suggests the down-regulation of *ELOVL2* induces lipid metabolism reprogramming in breast cancer and contributes to their malignant phenotypes.

## Introduction

Since the discovery of the Warburg effect, in which cancer cells preferentially utilize glycolysis and lactate fermentation even in the presence of oxygen, many other metabolic alterations, such as glutaminolysis and serine/glycine biosynthesis, have been established (1–3). These altered metabolic pathways exhibit significantly important functions in cancer initiation, progression, and drug resistance. Thus, they are considered to be attractive targets for cancer diagnostic and therapeutic development (4–6). Recently, aberrant lipid metabolism has been identified in different types of cancer and associated microenvironments, and awareness of the multifaceted role of lipid metabolism in cancer has rapidly increased (7–10). Of note, a universal feature of cancer is the enhanced lipid biosynthesis, which is reflected in the activation of several key lipogenic factors, including ACLY, ACACA, FASN, and SCD, as well as the increased activity of the SREBP family of transcription factors (9, 11, 12). SREBPs are key regulators of fatty acid and cholesterol biosynthesis (9). Importantly, SREBP-mediated lipogenesis supports the processes of proliferation, survival, maintenance, and metastasis during cancer progression. Thus, lipogenic molecules are widely considered attractive targets for the development of anti-cancer therapies (9, 11, 12).

Despite the fact that altered lipid metabolism has long been recognized to play an important role in both the aggressiveness and progression of cancer, precise characterizations of the essential components in lipid metabolic networks, such as the compositional changes of lipids, are lacking (7–9). Recent evidence suggests that the rewiring in lipid metabolism, such as in the acyl-CoA synthetase/stearoyl-CoA desaturase network, promotes the migration and invasion of cancer cells through the epithelial-mesenchymal transition (EMT) (13). EMT is a complex biological process that is regulated in a multiplex manner, from gene expression to post-translational modifications along with the contributions of various microenvironemental factors (14, 15). Several fatty acid regulators are associated with poor survival rates in an EMT context (16). As EMT is associated with cancer progression and metastasis, examining EMT-related alterations in lipid metabolism may provide additional insights into the mechanisms underlying the metastasis and drug resistance of cancer (17). Moreover, the roles of these pathways in the aggressiveness and progression of cancer have not been clearly identified. This may be due, in part, to the lack of high-throughput and comprehensive approaches to characterize the complex mammalian lipidomes, which consists of thousands of unique structures and derives from a wide range of different lipid classes and fatty acyl chain variants (18). Furthermore, complex lipid networks that are regulated by hundreds of enzymes and lipid species are usually difficult to be appropriately investigated. To overcome these limitations, lipidomics, driven by the rapid development of mass spectrometry, has been developed and used to achieve outstanding results in recent years (19–23). In addition, lipidomic analysis can be complemented by the integration of other functional -omics data, such as transcriptomics, or stable isotope-assisted metabolic labeling to generate a sophisticated framework to get deeper insights into biological processes (24, 25). This combined approach can facilitate mechanistic understanding of the crucial components of the atypical lipid metabolism present in various disease states (22). Furthermore, multi-layered omics analysis is certainly essential for the discovery phase of a mechanistic study due to the ability to provide a big picture into the phenotypes of interest (26, 27).

Herein, we aimed to characterize the alterations in the breast cancer lipidome during the EMT malignant transformation of breast cancer. Thus, a mammosphere-based EMT model, which has been found to increase tumor-initiation, progression, and drug resistance of cancer cells, was utilized for the investigation (28). In the discovery phase, we applied a comprehensive lipidomics approach guided by transcriptomics data to investigate the alterations of lipid metabolism of the spheroid cancer cells. The spheroid-culture system exhibiting EMT properties was established and well validated from our previous work (29). As a result, we observed a unique aberrant pattern regarding the lipidome of EMT-induced breast cancer cells. The findings were then extended by an independent experiment specifically targeted on fatty acid compositions of the cells. Further mechanistic study discovered a novel process that the depletion of *ELOVL2* increased the malignant phenotypes of breast cancer. Finally, *ELOVL2* was later found to be down-regulated in the more aggressive phenotypes of breast cancer among 51 cell lines and associated with the clinically worse outcome of breast cancer patients.

## Materials and Methods

### Adherent Cell Culture

The MCF-7 breast cancer cell line was purchased from the American Type Culture Collection (ATCC, Manassas, VA, USA). The cells were maintained using DMEM with 10% fetal bovine serum (Life Technologies, Carlsbad, CA, USA) supplemented with 100 μg/mL streptomycin, and 100 μg/mL penicillin.

### Mammosphere Cell Culture

Single-cell suspensions of MCF-7 cells were seeded into an ultralow adherence dishes (Corning, Corning, NY, USA) at a density of 5 × 10^5^ cells/mL in DMEM/F12 containing 1 × B27 supplement (Life Technologies) supplemented with 20 ng/mL basic fibroblast growth factor (R&D Systems), 20 ng/mL recombinant epidermal growth factor (Life Technologies), 100 U/mL penicillin, and 100 μg/mL streptomycin. Each culture was fed twice a week and subjected to a biweekly trypsinization and dissociation using a 23-gauge needle. Individual cells were pelleted and suspended in mammosphere medium in ultralow adherence dishes at 5 × 10^5^ cells/mL.

### Transcriptomic Analysis

The normalized gene expression data of the mammosphere cell culture and adherent cell culture was gathered from our previous investigation via ArrayExpress (E-MTAB-3860) (30). Log_2_ fold-changes of targeted genes between Sphere and Adherent were extracted and visualized.

### Western Blot Analysis

For the preparation, cells were lysed in 0.5% NP-40/Tris M2 buffer (31). A standard protocol for Western blot experiment of cell extracts were used with 12% sodium dodecyl sulfate-polyacrylamide gel electrophoresis (SDS-PAGE) and visualized by enhanced chemiluminescence (ECL; Amersham International, Amersham, UK). The ELOVL2 antibody was from Abcam (Cambridge, UK), the β-actin antibody was from Sigma-Aldrich (St. Louis, MO, USA).

### Lentiviral shRNA Experiments

MISSION shRNA plasmids targeting the coding region or 3′ UTR of *ELOVL2* mRNA (NM_017770) and non-targeted control sequences (NM_027088) were purchased from Sigma-Aldrich. Lentiviral plasmids were transfected into 293TN cells (LV900A-1, System Biosciences, Mountain View, CA, USA) using Lipofectamine 2000 (11668019, Invitrogen, Carlsbad, CA, USA). Pseudoviral particles were collected 2 days after the transfection of lentiviral plasmids and used to infect MCF-7 cells in the presence of polybrene (8 μg/mL). Infected cells were selected with puromycin beginning 2 days after infection, and *ELOVL2* knockdown was confirmed by immunoblotting.

### Reverse Transcription Polymerase Chain Reaction (RT-PCR)

Primer sequences used for PCR amplification were β*-actin* (sense 5′-GTGGGGCGCCCCAGGCA-3′, anti-sense 5′-CTCCTTAATGTCACGCACGAT-3′), *ELOVL2* (sense 5′-CTCTCCACTTGGGAAGGAGG-3′, anti-sense 5′-AGATGAGACAACCGAAGGGG-3′), *ELOVL3* (sense 5′-GGTCCTTCTGCCTTGCAATCTTCAG-3′, anti-sense 5′-TTCACTGGCTCTTGGTCTTGGCTTTGAC-3′), *PECR* (sense 5′-ATGGAGGAGGCCAGTTTCTT-3′, anti-sense 5′-AGGAAGCAGACCACAGAGGA-3′), PTPLB (sense 5′-GTCCGAGCATACCTGGCTAA-3′, anti-sense 5′-ACTCCCATTGGGTACAGCAC-3′), *SCD* (sense 5′-TTGGAGAAGCGGTGGATAAC-3′, anti-sense 5′-AAAAATCCCACCCAATCACA-3′), *SREBP1* (sense 5′-CTTCTGACAGCCATGAAGACAG-3′, anti-sense 5′-GTGTTGCAGAAAGCGAATGTGG-3′), and *SREBP2* (sense 5′-AACAGCTGTGTAGCTCCTTTCC-3′, 5′-ATATCAAAGGCTGCTGGATGAT-3′). RNA was extracted using RNeasy (Qiagen, Valencia, CA, USA). A total of 200 ng of RNA from each sample was used for cDNA synthesis with a reverse transcriptase kit (Invitrogen). Equal amounts of cDNA product were used for PCR, which was performed with Taq DNA polymerase (Takara, Kyoto, Japan). The final PCR products were resolved with a 1.5% agarose gel and stained with ethidium bromide.

### Wound Healing Assay

Scratched cells were cultured for 72 h in total, and representative images were taken by a phase-contrast microscope at 0, 24, 48, and 72 h. Under the microscope, the distance of the wound was measured at 5 different points to quantify the recovery rate. Recovery rates are expressed as the mean ± SEM from duplicate determinations of three independent experiments for each case.

### Colony Formation Assay

Cancer cells were plated with 500, 2,000, and 5,000 cells per well and maintained for 2 weeks. The medium was later discarded. Then, each well was washed carefully twice with PBS, and formed colonies were fixed in methanol for 10 min and stained with crystal violet solution for visualization.

### LC-MS-based Cellular Lipid Profiling

Two-hundred microliter of 0.9% NaCl solution was added to the cell pellet, which was immediately homogenized for 30 s on ice (sonication for 3 s and intermission for 3 s) using the Vibra-Cell ultrasonic homogenizer (Sonics, Newtown, CT, USA). Lipid class-specific internal standards consisting of 400 ng of LPC (17:0), PC (17:0/17:0), PE (17:0/17:0), and Cer (18:1/17:0), and 2,000 ng of TG (17:0/17:0/17:0) were spiked into the remaining homogenate, and cellular lipids were extracted using 600 μL of a chloroform-methanol mixture (2:1, v/v). After vortexing the mixture for 2 min, allowing it to stand for 30 min at 20°C, and centrifuging the sample for 5 min at 16,000 × g, the lower organic phase was collected into a glass vial and injected for LC-MS analysis using an Agilent 1260 HPLC and 6530 QTOF-MS system (Agilent Technologies) equipped with an electrospray ion source with Agilent Jet Stream Technology. In detail, lipids were separated on a reverse-phase C18 SPP column (Brownlee SPP C18, 2.7 μm, 2.1 mm × 75 mm; PerkinElmer, Branchburg, NJ, USA) at 50°C with a gradient elution of mobile phase A (water, 1% 1 M ammonium acetate, and 0.1% formic acid) and mobile phase B (1:1 acetonitrile/2-propanol (v/v), 1% 1 M ammonium acetate, and 0.1% formic acid). Mobile phase A was used at a concentration of 65%, which was increased to 20% for 8 min, decreased to 0% for 14 min, and then maintained for 14 min with a flow rate of 0.4 mL/min. The QTOF-MS was operated in both positive and negative ESI modes. The MS and MS/MS (operated at 30 eV of fragmentation energy) spectra were used for structure identification and the quantification of lipids. The data processing for peak extraction and alignment was conducted as described previously (32). The lipid structure was identified based on an initial high-throughput lipid matching procedure using SimLipid (PremierBiosoft International, Palo Alto, CA, USA), followed by successive manual confirmations. The identified peaks were normalized by their class-specific internal standards and protein quantity for composition analysis.

### GC-MS-based Cellular Esterified Fatty Acid Profiling

Two-hundred μL of 0.9% NaCl solution was added to the cell pellet, which was immediately homogenized for 30 s on ice (sonication for 3 s and intermission for 3 s) using a Vibra-Cell ultrasonic homogenizer (Sonics). As an internal standard of conjugated fatty acids, 2.5 μg PC (17:0/17:0) or 5 μg C23:0 fatty acid methyl ester (FAME) was spiked into the sample. The methylation of conjugated fatty acid was performed as previously described (33). Two milliliters of 0.4 M KOH in MeOH was added to the homogenate, vortexed for 30 s, and incubated at 20°C for 10 min. Then, the FAMEs were extracted twice by liquid-liquid extraction using 2 mL of hexane. FAMEs containing a hexane layer were evaporated using a nitrogen purge and re-dissolved in 100 μL of hexane for GC/MS analysis using a Shimadzu QP2010A gas chromatography coupled to a GC MS-QP2010 mass spectrometer (Kyoto, Japan). In the chromatographic system, a DB-5 capillary column (30 m × 0.25 mm i.d., film thickness 0.25 μm) was used. The temperature gradient initially increased from 150 to 240°C at a rate of 3°C per min, and from 240 to 300°C at a rate of 5°C per min, and then was maintained at 300°C for 5 min. Sample injection was performed in splitless mode with 1.0 μL of sample injected. For MS analysis, the ion source and interface temperature were set to 200 and 300°C, respectively. The data was acquired in the full scan mode (30–600 m/z) under 70 eV of ionization voltage. FAMEs were identified by retention time and EI-MS spectrum matching with authentic FAME standards. Relative FAME compositions were calculated using the relative response factors of internal standard peaks (C17:0 FAME or C23:0 FAME) (Supplementary Table 1).

### Principal Component Analysis and Partial Least Squares-Discriminant Analysis

Principal component analysis (PCA) and partial least squares-discriminant analysis (PLS-DA) of all identified lipids was conducted and visualized using MetaboAnalyst 4.0 (34). Before the analysis, the data was quantile-normalized, log-transformed and scaled using pareto scaling method. Variable importance on projection (VIP) score of the first component from optimal PLS-DA model was extracted. Any features with a VIP score of one or higher are considered important features of the PLS-DA model.

### Pathway Enrichment Analysis

Gene Set Enrichment Analysis (GSEA) was utilized to conduct pathway analysis for our pre-defined set of genes with regard to the differences of two phenotypes of interest (35).

### Bioinformatics Based Survival Analysis

To evaluate the association between *ELOVL2* gene expression and any corresponding event-free (AE) or metastatic relapse-free (MR) survival rates, we conducted several meta-analyses using Breast Cancer Gene-Expression Miner version 4.0 (bc-GenExMiner v4.0, http://bcgenex.centregauducheau.fr/) (36, 37) with the patients divided into two groups expressing high or low level of *ELOVL2*. GEPIA was used to secondarily analyze the overall survival of all-type cancer patients in The Cancer Genome Atlas (TCGA) cohort with respect to the expression of *ELOVL2* (38). We were waived from a full ethical application for bioinformatics analysis by the Seoul National University Institutional Review Board.

### Statistical Analysis

Continuous data is expressed as the mean and standard error of the mean (SEM) and was analyzed using Student's two-tailed *t*-test unless otherwise stated. A value of 0.05 was considered to be the cut-off for the significance in statistical tests except otherwise mentioned. For high-dimensional data analysis, an adjusted *P*-value (false discovery rate) of 0.1 was applied for all multiple hypothesis tests. Gene expression with different grade comparison of breast cancer patients was conducted using Dunnett-Tukey-Kramer's test.

## Results

### EMT-induced Breast Cancer Cells Showed Distinctive Alterations in Lipid Composition

**Figure 1:**
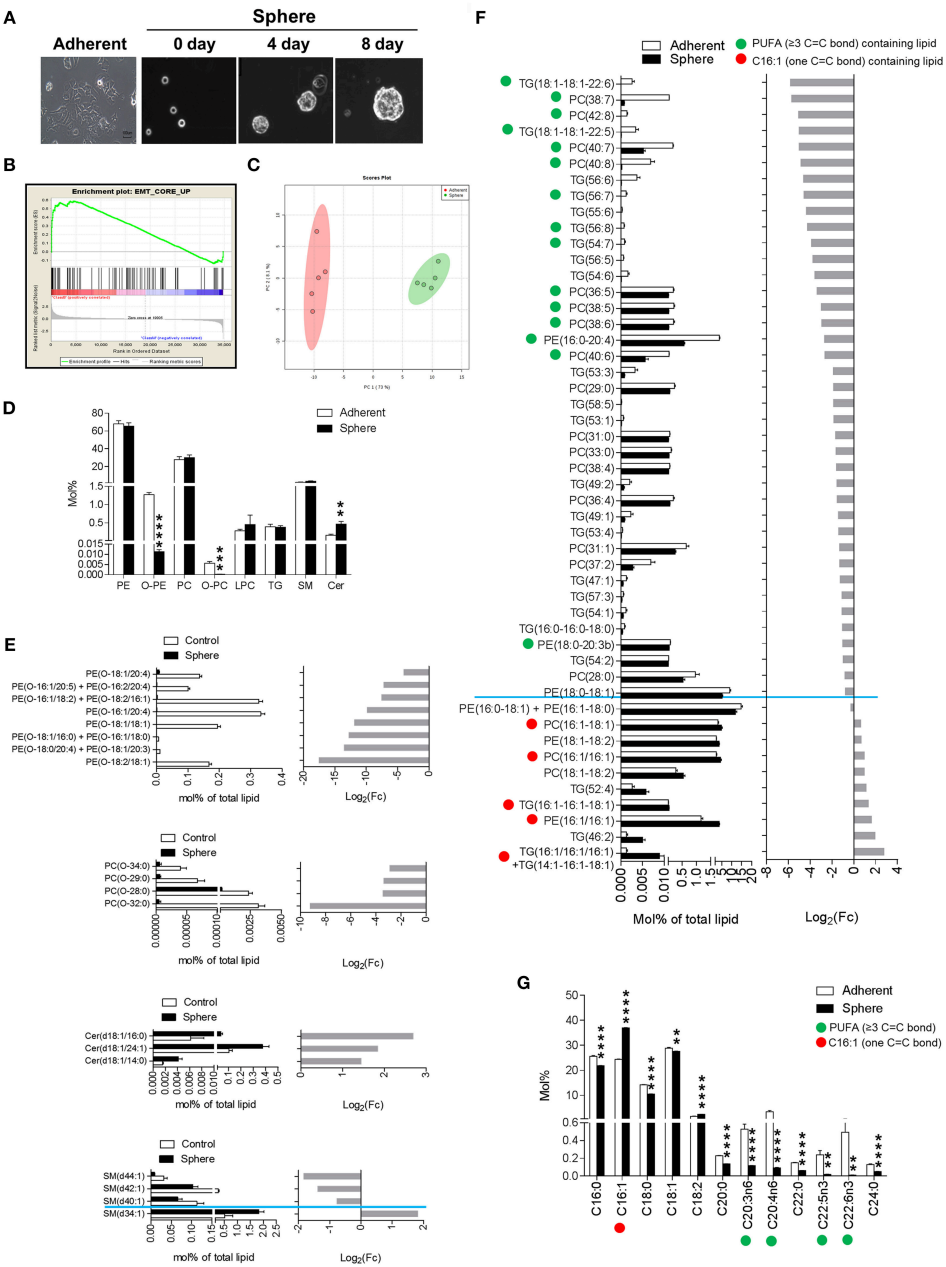
Epithelial-mesenchymal transition (EMT) phenotypic lipidomic changes of mammosphere-cultured MCF-7 cells. **(A)** Mammosphere cultures of cancer cells (Sphere). Representative images of day 0, 4, and 8 of the cultured cells, which were cultured in mammosphere-culture media, were taken daily with a phase-contrast microscope. **(B)** EMT core gene enrichment analysis. Expression of the EMT-related gene signature that was significantly enriched in Sphere. **(C)** Principal component analysis shows a clear tendency of difference in the lipid compositions between Sphere and Adherent. **(D)** Liquid chromatography-mass spectrometry (LC-MS)-based lipid class composition differences between Adherent and Sphere. Data is presented as the mean ± standard error of mean (SEM) (*n* = 5); ^**^P < 0.01; ^***^P < 0.001; and ^****^P < 0.0001, determined by Student's two-tailed *t*-test. **(E)** Altered individual lipid species compositions of phosphatidylcholine (PC), phosphatidylethanolamine (PE), and triglyceride (TG). The significantly altered individual lipid species compositions of PC, PE, and TG between Adherent and Sphere acquired by LC-MS-based lipid profile. Lipids containing C16:1, a kind of monounsaturated fatty acid (one C=C bond, red circle), and lipids containing polyunsaturated fatty acids (≥3 C=C bonds, green circle) are denoted. Data is presented as the mean ± SEM (*n* = 5). **(F)** Alteration of the fatty acid compositions. C16:1, a kind of monounsaturated fatty acid (one C=C bond, red circle), and polyunsaturated fatty acids (≥3 C=C bonds, green circle) are denoted. Data is presented as the mean ± SEM (*n* = 5). (**G**) Altered individual lipid species compositions of PE-O, PC-O, Cer, and SM. The significantly altered individual lipid species compositions of PE-O, PC-O, Cer, and SM between Adherent and Sphere acquired by LC-MS based lipid profile; ^**^P < 0.01 and ^****^P < 0.0001, determined by Student's two-tailed t-test.

We investigated the alterations in lipid metabolism of EMT-induced breast cancer cells by comparing them with the controls. To induce EMT, we adopted our previously established mammosphere culture system (29). As shown in Figure 1A, cancer cells exhibited tightly aggregated spheroids within 7–8 days of culture. After that, spheroids were dissociated, and then individual cells were re-suspended in sphere medium for 2 months. To identify differentially expressed genes between adherent cells (Adherent) and spheroid cells (Sphere), we performed a transcriptome analysis with the processed data (30). The highly different expressed genes between two conditions were used for gene set enrichment analysis (GSEA). As a result, the expression of EMT-related gene signature (EMT_CORE_UP, *n* = 91) was significantly enriched in sphere-cultured cells (enrichment Score = 0.588, Figure 1B). Under these conditions, we also performed comparative genomic hybridization (CGH) analysis between Adherent and Sphere cells to assess genome copy number abnormalities. It turned out that cells from the two groups exhibited no significant differences in copy number variation (Supplementary Figure 1). Collectively, the spheroid phenotype exhibited EMT properties and it might come from the post-translational modification processes.

Next, to investigate the association between global lipid changes and EMT-induced cancer cells, we acquired the lipidomes of Sphere and Adherent cells using liquid chromatography-mass spectrometry (LC-MS). Using an internal spectral library, which consisted of MS and tandem mass spectrometry (MS/MS) spectra of class-specific lipid standards (Supplementary Figure 2A), an initial high-throughput lipid matching procedure was followed by successive manual confirmations to identify lipid structures and remove false positives from the automatic identification system (Supplementary Figure 2B). In total, 123 lipid species were identified with high confident, belonging to eight classes of lipids: lysophosphatidylcholine (LPC), phosphatidylcholine (PC), ether-linked phosphatidylcholine (PC O-), phosphatidylethanolamine (PE) and ether-linked phosphatidylethanolamine (PE O-), triglyceride (TG), sphingomyelin (SM), and ceramide (Cer). A comprehensive MS and MS/MS spectrum based identification approach is given in Supplementary Figure 3. Interestingly, there was a huge difference of the lipid composition (73 of 123) between Sphere and Adherent (*P* < 0.05 and FDR < 0.10) (Supplementary Table 2). The principal component analysis (PCA) shows a clear tendency of overall difference in the lipid composition between two groups (Figure 1C). Following PLS-DA once again confirmed distinct lipidomes between two groups with 39 lipid species had VIP score of 1 or higher (*R*^2^ = 1.00, *Q*^2^ = 0.98, Supplementary Figure 4 and Supplementary Table 3). Specifically, Cer was increased in Sphere cells, whereas the ether-linked glycerophospholipids PC O- and PE O- were decreased (Figure 1D). Our observations were extended by investigating the fatty acid compositions of lipids according to the follow-up GC/MS analysis results. Indeed, the lipids containing C16:1 was increased while lipids with PUFA moieties (≥3 C=C bonds) were dramatically decreased in Sphere cells. A deeper look of the alteration patterns of PC O-, PE O-, Cer, and SM are given in Figure 1E. For instance, within the SM class, SM (d34:1) was decreased in Sphere cells, while SM (d40:1), SM (d42:1), and SM (d44:1), which consist of longer chain fatty acids, were increased. In addition, we observed global changes with specific patterns dependent on the type of fatty acid residues of individual lipids contained within PC, PE, and TG although the total compositions were not changed (Figure 1F). PE, PC, and TG species containing C16:1, which is a type of mono-unsaturated fatty acid (MUFA, one C=C bond) moiety, were elevated in Sphere cells. In contrast, lipids containing a highly unsaturated polyunsaturated fatty acid (PUFA) moiety (≥3 C=C bonds) were dramatically reduced in Sphere cells (Figure 1G). Inclusively, the results indicated a distinctive difference in the lipidome and especially fatty acid compositions between adherent and sphere culture-based transformed cancer cells that underwent the EMT process.

### Fatty Acid Metabolic Gene Expression Is Altered During Sphere Culture of Cancer Cells

**Figure 2:**
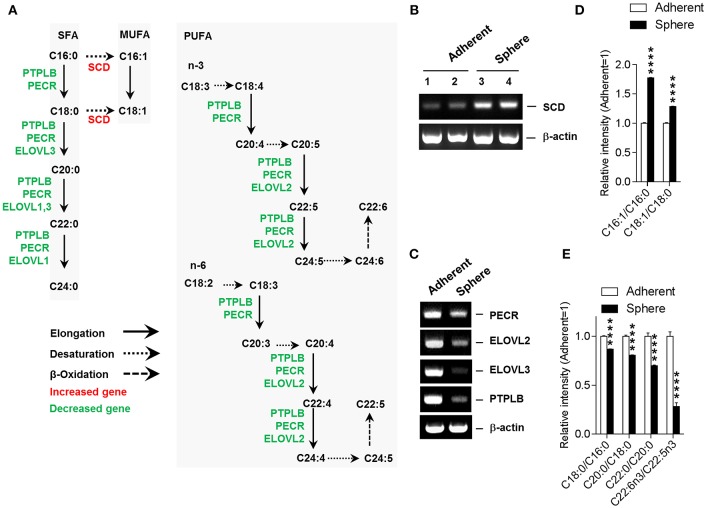
Integrative unsaturated fatty acid biosynthesis pathway. **(A)** Gene expression pattern for unsaturated fatty acid biosynthesis pathway. Altered expression of genes involved in unsaturated fatty acid biosynthesis is denoted (increased, red; decreased, green). **(B)** Up-regulation of *SCD* in sphere cells. Reverse transcription-polymerase chain reaction (RT-PCR) shows the up-regulation of *SCD* in sphere-cultured MCF-7 cells. **(C)** Downregulation of *PECR, ELOVL2, ELOVL3*, and *PTPLB*. Sphere-cultured MCF-7 cells showed down-regulated *PECR, ELOVL2, ELOVL3*, and *PTPLB* mRNA. **(D)** Up-regulation of mono-unsaturated fatty acid (MUFA) to saturated fatty acid (SFA) ratios in sphere cells. Sphere-cultured MCF-7 cells showed up-regulated C16:1/C16:0 and C18:1/C18:0 **(E)** Downregulation of elongation associated product to substrate fatty acid ratios in sphere cells. Sphere-cultured cells showed down-regulated C18:0/C16:0, C20:0/C18:0, C22:0/C20:0, and C22:6n3/C22:5n3. **(D,E)**. Data is presented as the mean ± SEM (*n* = 5). ^****^P < 0.0001, determined by Student's two-tailed *t*-test.

To gain insight into alterations in the fatty acid compositions of spheroid cells, we analyzed the altered gene expression patterns of associated metabolic enzymes involved in the unsaturated fatty acid biosynthesis pathway (Supplementary Table 4). Coinciding with the altered compositions of lipids and fatty acids, the gene expression results revealed an enhanced MUFA biosynthesis and depleted PUFA biosynthesis in Sphere cells (Figure 2A). *SCD*, which has a central role in the production of MUFA from saturated fatty acid (SFA), was up-regulated in Sphere cells. Conversely, the gene expression level of *PTPLB, PECR, ELOVL2*, and *ELOVL3*, which are all involved in fatty acid elongation and PUFA biosynthesis, were down-regulated in Sphere cells. The mRNA expression level of fatty acid regulatory genes were further confirmed by reverse transcription-polymerase chain reaction (RT-PCR) (Figures 2B,C). Moreover, consistent with these regulatory gene expression patterns, the product to substrate ratios of fatty acids confirmed increases in the MUFA to SFA ratios and decreases in the fatty acid (FA) (+2 carbons) to FA ratios in Sphere cells. Last but not least, the ratio of C22:6n3 (docosahexaenoic acid, DHA) to C22:5n3 was dramatically reduced in Sphere cells (Figures 2D,E).

### ELOVL2 Silencing Alters Fatty Acid Metabolism in Breast Cancer Cells And Promotes Malignant Phenotypes

**Figure 3:**
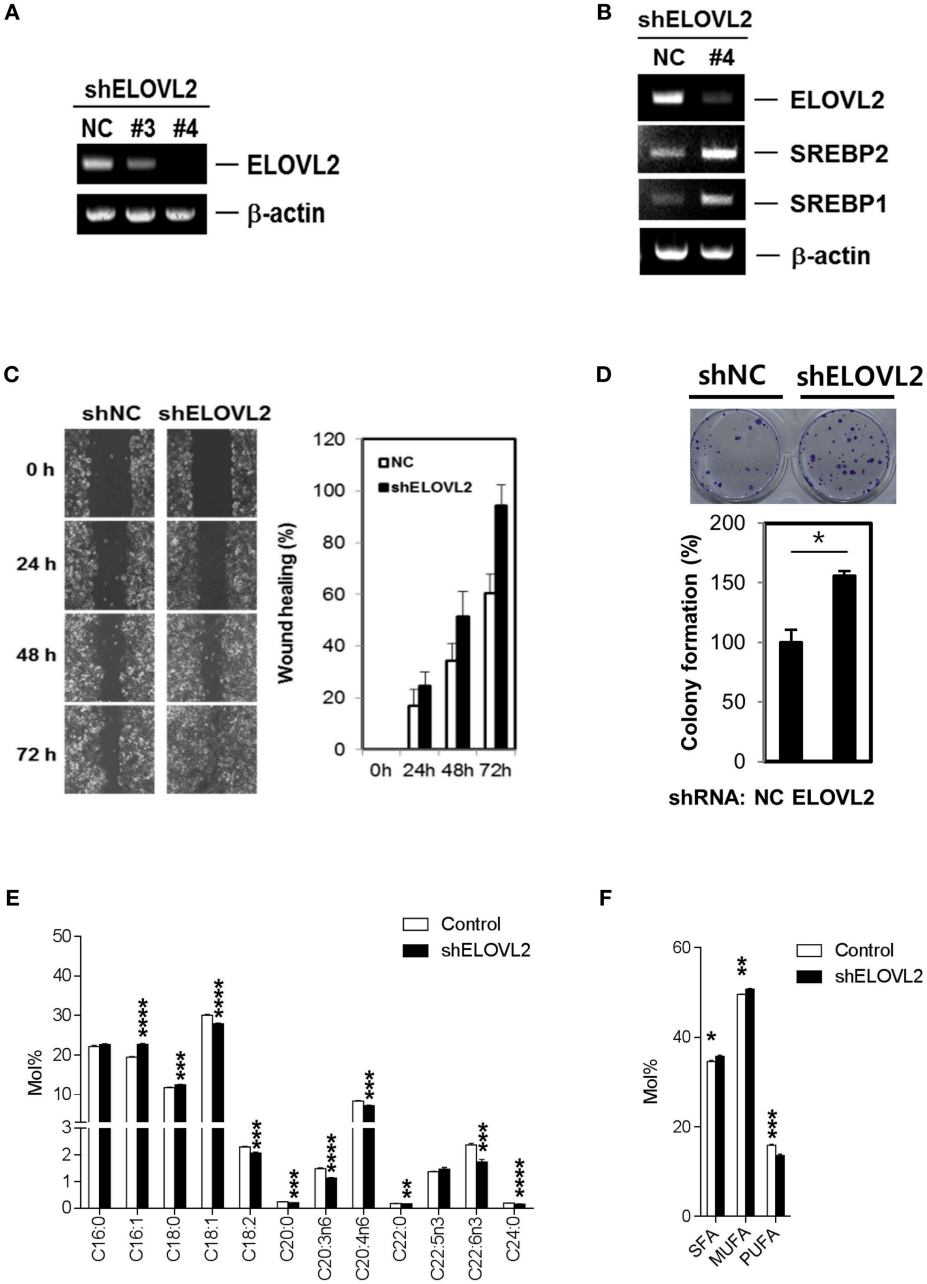
*ELOVL2* silencing alters fatty acid metabolism in breast cancer cells and promotes malignant phenotypes. **(A)** Knockdown of *ELOVL2*. MCF-7 cells with stable short-hairpin RNA (shRNA) *ELOVL2* integration were analyzed by RT-PCR. **(B)** Inhibition of *ELOVL2* expression increased *SREBP1* and *SREBP2* mRNA levels. MCF-7 cells with stable shRNA *ELOVL2* integration were analyzed by RT-PCR to examine *SREBP1* and *SREBP2* mRNA level. **(C)** Reduced *ELOVL2* gene expression increased wound healing activity. MCF-7 cells with stable shRNA *ELOVL2* integration were evaluated to determine the effects on motility using a wound-healing assay. All data shown are representative of three independent experiments. **(D)** Reduced *ELOVL2* gene expression increased colony forming activity. ^*^P < 0.05; ^**^P < 0.01; ^***^P < 0.001; and ^****^P < 0.0001, determined by Student's two-tailed *t*-test. **(E)** Distinct alteration of individual fatty acid compositions in lipids. Data is presented as the mean ± SEM (*n* = 5); ^*^P < 0.05; ^**^P < 0.01; ^***^P < 0.001; and ^****^P < 0.0001, determined by Student's two-tailed *t*-test. **(F)** Alteration of SFA, MUFA, and poly-unsaturated fatty acid (PUFA) (≥3 C=C bonds) compositions in lipids. Data is presented as the mean ± SEM (*n* = 5); ^*^P < 0.05; ^**^P < 0.01; ^***^P < 0.001, determined by Student's two-tailed *t*-test.

Although the biological significance of active fatty acid biosynthesis in cancer has been widely studied (9, 11, 12), a systematic investigation with regard to the roles of altered PUFA biosynthesis pathways in breast cancer is lacking. Several lines of evidence have suggested that ELOVL2 is related to the regulation of lipid metabolism via the production of DHA (39). DHA contributes to lipid homeostasis by inhibiting SREBPs (39–41), key transcription factors that enhance cancerous fatty acid biosynthesis by activating several lipogenic enzymes (42). Additionally, ELOVL2 has not been known to be involved in any hallmarks of cancer with respect to our text mining result using Cancer Hallmark Analytics Tool (43). Hence, to further examine the roles of ELOVL2 in breast cancer cells, we introduced short hairpin RNAs (shRNAs) to silence *ELOVL2* in breast cancer cells (Figure 3A). Interestingly, the downregulation of *ELOVL2* resulted in the increased expression of *SREBP1* and *SREBP2* (Figure 3B). *ELOVL2*-depleted cancer cells exhibited a more malignant phenotype than that of the control group. Specifically, ELOVL2 knockdown cells showed a higher migration rate in wound healing assay (Figure 3C) and higher colony formation (Figure 3D). Furthermore, *ELOVL2* knockdown led to significant changes in the lipid compositions of fatty acids, not only decreasing DHA but also increasing SFA and MUFA (Figures 3E,F). These results suggest that the downregulation of *ELOVL2* may induce lipogenesis by reducing DHA and enhancing the SREBP pathway.

### Associations Between ELOVL2 Down-Regulation and Malignant Phenotype in Breast Cancer

**Figure 4:**
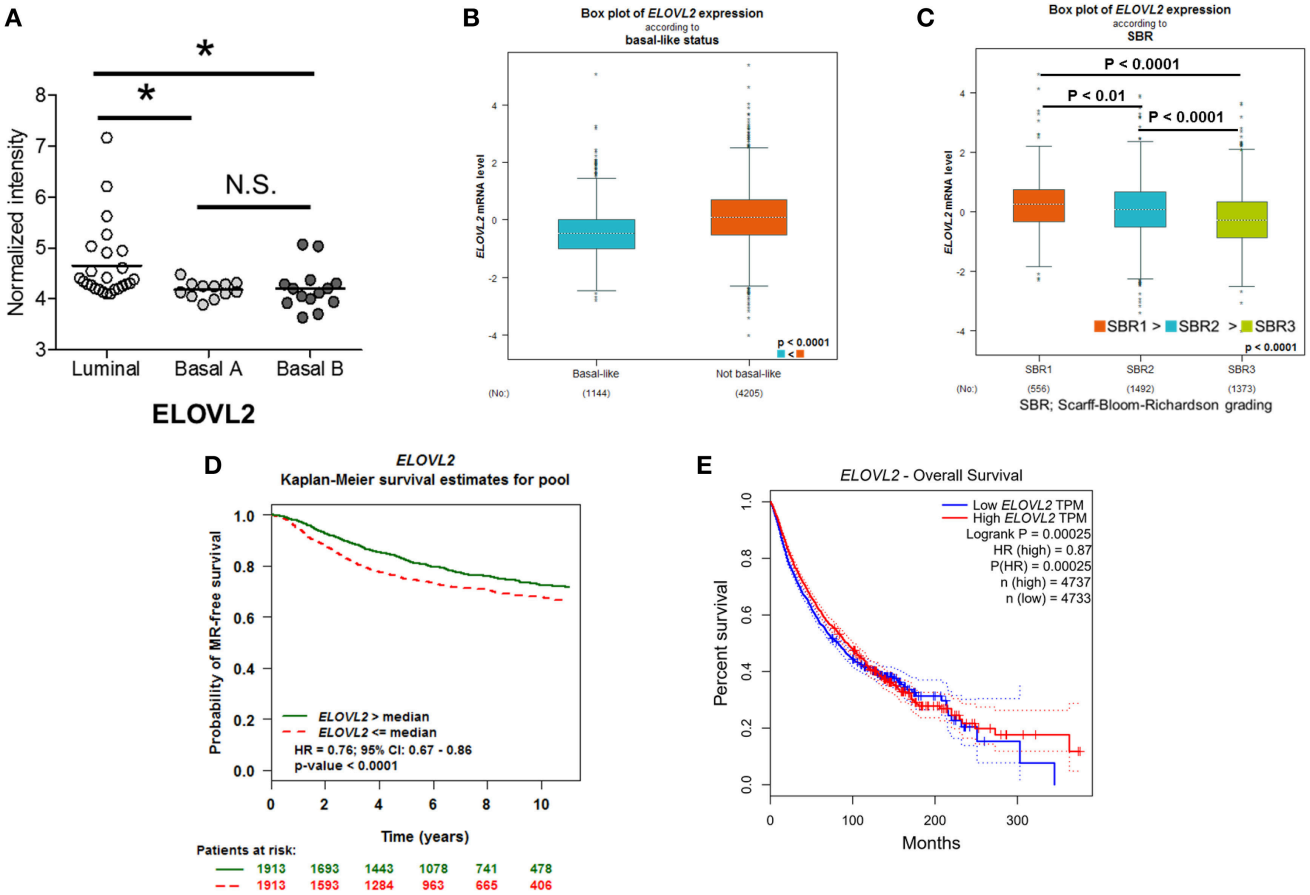
Reduced *ELOVL2* gene expression is associated with a more malignant phenotype in breast cancer. **(A)** *ELOVL2* gene expression in different types of breast cancer cell lines. The analysis was performed using the previous microarray datasets, which consisted of 51 breast cancer cell lines (44). ^*^P < 0.05, determined by Wilcoxon rank-sum test. **(B)** *ELOVL2* gene expression in patients with basal and non-basal like breast cancer types. The *P*-value was determined by Welch's test. **(C)** *ELOVL2* gene expression in patients with different grades of breast cancer from the Scarff-Bloom-Richardson grading system. The *P*-value was determined by Dunnett-Tukey-Kramer's test. *ELOVL2* gene expression-dependent Kaplan-Meier survival estimate of **(D)** metastasis-relapse (MR) free survival in breast cancer patients. **(E)** Worse overall survival of the patients with lower level of *ELOVL2* in pan-cancer analysis.

Our findings regarding the contribution of ELOVL2 to the malignant phenotypes of breast cancer were further explored by two large-scale analyses. First, the analysis of a previous microarray dataset consisting of 51 breast cancer cell lines (44) showed a significant attenuation of *ELOVL2* expression in basal-like breast cancer cell lines associated with EMT (Basal A and Basal B) (45) compared to luminal breast cancer cell lines (Figure 4A). Moreover, *ELOVL2* expression was consistently reduced in breast cancer patients with basal-like status (*N* = 1,144) compared to that in patients with non-basal-like status (*N* = 4,205) (Figure 4B). Lower levels of *ELOVL2* expression were associated with a higher grade of breast cancer and lower metastatic relapse-free survival over a 10-year period (Figures 4C,D). By way of addition, the overall survival of the patients with lower level of *ELOVL2* (by median) in a pan-cancer analysis was worse than that of the counterpart group (Figure 4E). Collectively, we demonstrated a strong evidence that the down-regulation of *ELOVL2* expression could be associated with the aggressiveness and progression of breast cancer and it was inferred that the tumor suppression mechanism of *ELOVL2* was associated with lipid metabolism (46).

## Discussion

The advancement of high-throughput technologies, especially omics has gained its popularity and become an important approach in biomedical sciences (6, 47, 48). Among them, lipidomics aims to explore the underlying mechanisms associated with metabolically related diseases, including cancer (49). Global lipidomics in combination with other omics technologies give us an unprecedented opportunity to explore the alterations of lipid metabolism at the systematic scale (50). Various classes and subclasses of lipids could be monitored at the same time and eventually being deeply investigated. Moreover, abnormal lipid metabolism is closely associated with a malignant cancer phenotype, and the activation of lipid biogenesis pathways contributes to cell transformation and cancer development (51, 52). As a proof of concept, a recent study utilized a multi-layered omics approach to investigate the metabolic programming of breast cancer cells with respect to EMT. The work demonstrated that β-catenin has an essential role in regulating metabolic network of mesenchymal cancer cells (27). In this study, by using a well-established spheroid-induced epithelial-mesenchymal transition (EMT) model and a comprehensive multi-omics approach, we were able to explore the aberrant of the lipidome of the breast cancer cells during the malignant transformation. It is of importance to mention that we provide a rule-based comprehensive MS and MS/MS spectrum identification protocol (Supplementary Figure 3). As a result, a large number of lipids were found to be altered. Indeed, Cer and longer chain fatty acid SM species were significantly increased in the spheroid cells. The aberrant sphingolipid metabolism has been found to be associated with the drug resistance. In addition, Cer and SM related regulatory processes are encompassed in the differentiation, proliferation, and apoptosis via various intrinsic and extrinsic pathways (53). By discovering the association between EMT and altered lipidome in breast cancer, we provide novel targets for new therapeutic development. Furthermore, the fatty acid moieties in the lipidome were significantly altered although there were no global change of PC, PE, and TG. Of note, enhanced fatty acid synthesis has been acknowledged as an important characteristics of cancer cells (52). However, the precise underlying regulatory mechanisms have yet to be determined. When extending the study on the fatty acid alterations, we indicated the simultaneous alteration of MUFA biosynthesis (activated) and PUFA biosynthesis (deactivated) pathways during the transformation of breast cancer cells. Activation of the MUFA pathway has been observed in several cancers and is suggested to represent a predictive marker (54, 55). Up-regulation of the MUFA synthesis enzyme, SCD, has been observed in several types of human cancers, and its gene expression is correlated with the malignant transformation of cancer and poor survival rates (56).

Notably, our findings implicate *ELOVL2* depletion in breast cancer progression. ELOVL2 is a critical enzyme in the biosynthesis of DHA (39), a functional PUFA inhibiting SREBPs by reducing their expression level or blocking the proteolytic process in biological systems (39, 57, 58). SREBPs induce various *de novo* lipogenic enzymes, which are essential in cancer progression (59, 60). ELOVL2 has recently found to be a critical pro-survival enzyme that help prevent the death and dysfunction of beta cell by glucolipotoxicity (61). Its silencing is associated with the decreased insulin secretion in rodent and human. This finding suggests that ELOVL2 may be involved in not only fatty acid metabolism but also in the regulation of glucose via insulin secretion (62). Here, we demonstrated that the downregulation of *ELOVL2* increases *SREBP1* expression in breast cancer cells and activates lipogenesis, which is associated with malignant phenotypes of breast cancer. Our findings also suggest that the therapeutic potential of DHA, a non-toxic natural essential fatty acid, and the underlying mechanism of SREBP regulation by DHA need to be evaluated by targeting lipogenesis of breast cancer. Finally, *ELOVL2* seems to be a novel prognostic biomarker of which its attenuation is significantly associated with a worse outcome of the cancer patients, including breast cancer. The use of *ELOVL2*, combining with current clinical biomarkers as well as cutting-edge statistical learning implementations, may improve the prognostic prediction for cancer patients (63).

Our study has several limitations need to be acknowledged. Although the main findings were supported by various experiments and clinical data from large cohorts, there was a lack of supportive evidence from a xenograft mouse model. The association between *ELOVL2* and EMT markers were also not revealed in the current work. Further studies are warranted to address the remaining limitations and extend our understanding on the role of *ELOVL2* in cancer.

In conclusion, we explored a global change on the lipidome of breast cancer during EMT as well as discovered the novel effects of *ELOVL2* attenuated gene expression in fatty acid biosynthesis, which may enhance the malignant phenotypes of breast cancer. The findings provide deeper insights into the roles of aberrant lipid metabolism during malignant transformation processes of breast cancer, especially the fatty acid and sphingolipid biosynthesis. Additionally, the down-regulated *ELOVL2* can be utilized as a prognostic biomarker for breast cancer patients. However, the ultimate mechanisms that are responsible for cancer-associated decreased expression of *ELOVL2* remain to be understood.

## Data Availability

Transcriptome data of MCF-7 breast cancer cell lines can be downloaded from ArrayExpress with the accession code of E-MTAB-3860. Normalized LC-MS/MS data of 126 lipids are given in the Supplementary Table 1. Other data supporting the findings of this study is available upon considerable request.

## Author Contributions

YK and J-HY performed experiments, analyzed and interpreted data, and wrote the first draft. NL participated in data collection, analysis and interpretation, and manuscript preparation. YK and NL proposed the lipid identification process used for this study. G-BK, S-JO, and H-JN performed *in vitro* experiments. SBL, HK, JH, WL, and SJL participated in data analysis and interpretation. SK, S-SH, and Y-SK conceived the study, interpreted data, supervised the study, and revised the manuscript. All authors read, critically discussed, and approved the final manuscript.

### Conflict of Interest Statement

The authors declare that the research was conducted in the absence of any commercial or financial relationships that could be construed as a potential conflict of interest.

## Abbreviations

ACACA: acetyl-CoA carboxylase
ACLY: ATP-citrate lyase
Cer: ceramide
CGH: comparative genomic hybridization
ELOVL2: elongation of very long-chain fatty acid 2
ELOVL3: elongation of very long-chain fatty acid 3
EMT: epithelial-mesenchymal transition
PC O-: ether-linked phosphatidylcholine
PE O-: ether-linked phosphatidylethanolamine
FASH: fatty acid synthase
FAME: fatty acid methyl ester
FDR: false discovery rate
GC-MS: gas chromatography-mass spectrometry
LPC: lysophosphatidylcholine
LC-MS: liquid chromatography-mass spectrometry
MS/MS: tandem mass spectrometry
MUFA: mono-unsaturated fatty acid
PC: phosphatidylcholine
PE: phosphatidylethanolamine
PECR: peroxisomal trans-2-enoyl-CoA reductase
PTPLB: protein tyrosine phosphatase-like member B
PUFA: polyunsaturated fatty acid
SCD: stearoyl-CoA desaturase
SFA: saturated fatty acid
SM: sphingomyelin
SREBP: sterol regulatory element-binding protein
TG: triglyceride.

## References

[1] DeNicola GM Cantley LC. Cancer's fuel choice: new flavors for a picky eater. Mol Cell. (2015) 60:514–23. 10.1016/j.molcel.2015.10.018 26590711 PMC4676726

[2] Liberti MV Locasale JW The warburg effect: how does it benefit cancer cells? Trends Biochem Sci. (2016) 41:211–218. 10.1016/j.tibs.2015.12.001 26778478 PMC4783224

[3] Eichenlaub T Villadsen R Herranz H Cohen SM. Warburg effect metabolism drives neoplasia in a drosophila genetic model of epithelial cancer. Curr Biol. (2018) 28:3220–8. 10.1016/j.cub.2018.08.035 30293715

[4] DeNicola GM Chen P-H Mullarky E Sudderth JA Hu Z Wu D. NRF2 regulates serine biosynthesis in non-small cell lung cancer. Nature Genetics. (2015) 47:1475–81. 10.1038/ng.3421 26482881 PMC4721512

[5] Pacold ME Brimacombe KR Chan SH Rohde JM Lewis CA Swier LJ A PHGDH inhibitor reveals coordination of serine synthesis and one-carbon unit fate. Nat Chem Biol. (2016) 12:452–8. 10.1038/nchembio.2070 27110680 PMC4871733

[6] Long NP Yoon SJ Anh NH Nghi TD Lim DK Hong YJ. A systematic review on metabolomics-based diagnostic biomarker discovery and validation in pancreatic cancer. Metabolomics. (2018) 14:109. 10.1007/s11306-018-1404-2 30830397

[7] DeBerardinis RJ Lum JJ Hatzivassiliou G Thompson CB. The biology of cancer: metabolic reprogramming fuels cell growth and proliferation. Cell Metab. (2008) 7:11–20. 10.1016/j.cmet.2007.10.002 18177721

[8] Hsu PP Sabatini DM. Cancer cell metabolism: warburg and beyond. Cell. (2008) 134:703–7. 10.1016/j.cell.2008.08.021 18775299

[9] Baenke F Peck B Miess H Schulze A. Hooked on fat: the role of lipid synthesis in cancer metabolism and tumour development. Dis Models Mech. (2013) 6:1353–63. 10.1242/dmm.011338 24203995 PMC3820259

[10] Sun L Suo C Li S-t Zhang H Gao P. Metabolic reprogramming for cancer cells and their microenvironment: beyond the warburg effect. Biochim Biophys Acta Rev Cancer. (2018) 1870:51–66. 10.1016/j.bbcan.2018.06.005 29959989

[11] Menendez JA Lupu R. Fatty acid synthase and the lipogenic phenotype in cancer pathogenesis. Nat Rev Cancer. (2007) 7:763–77. 10.1038/nrc2222 17882277

[12] Ackerman D Simon MC. Hypoxia, lipids, and cancer: surviving the harsh tumor microenvironment. Trends Cell Biol. (2014) 24:472–8. 10.1016/j.tcb.2014.06.001 24985940 PMC4112153

[13] Sánchez-Martínez R Cruz-Gil S de Cedrón MG Álvarez-Fernández M Vargas T Molina S. A link between lipid metabolism and epithelial-mesenchymal transition provides a target for colon cancer therapy. Oncotarget. (2015) 6:38719–36. 10.18632/oncotarget.5340 26451612 PMC4770732

[14] Simeone P Trerotola M Franck J Cardon T Marchisio M Fournier I. The multiverse nature of epithelial to mesenchymal transition. Semin Cancer Biol. (2018). 10.1016/j.semcancer.2018.11.004. [Epub ahead of print] 30453041

[15] Stefania DD Vergara D. The many-faced program of epithelial–mesenchymal transition: a system biology-based view. Front Oncol. (2017) 7:274. 10.3389/fonc.2017.00274 29181337 PMC5694026

[16] Nath A Chan C. Genetic alterations in fatty acid transport and metabolism genes are associated with metastatic progression and poor prognosis of human cancers. Sci Rep. (2016) 6:18669. 10.1038/srep18669 26725848 PMC4698658

[17] Lamouille S Xu J Derynck R. Molecular mechanisms of epithelial–mesenchymal transition. Nat Rev Mol Cell Biol. (2014) 15:178–96. 10.1038/nrm3758 24556840 PMC4240281

[18] Muro E Atilla-Gokcumen GE Eggert US. Lipids in cell biology: how can we understand them better? Mol Biol Cell. (2014) 25:1819–23. 10.1091/mbc.E13-09-0516 24925915 PMC4055261

[19] Wenk MR. The emerging field of lipidomics. Nat Rev Drug Discov. (2005) 4:594–610. 10.1038/nrd1776 16052242

[20] Wenk M Lipidomics R. New tools and applications. Cell. (2010) 143:888–95. 10.1016/j.cell.2010.11.033 21145456

[21] Shevchenko A Simons K. Lipidomics: coming to grips with lipid diversity. Nat Rev Mol Cell Biol. (2010) 11:593–8. 10.1038/nrm2934 20606693

[22] Watson AD. Thematic review series: systems biology approaches to metabolic and cardiovascular disorders. lipidomics: a global approach to lipid analysis in biological systems. J Lipid Res. (2006) 47:2101–11. 10.1194/jlr.R600022-JLR200 16902246

[23] Sundaram S Žáček P Bukowski MR Mehus AA Yan L Picklo MJ. Lipidomic impacts of an obesogenic diet upon lewis lung carcinoma in mice. Front Oncol. (2018) 8:134. 10.3389/fonc.2018.00134 29868466 PMC5958182

[24] S.-Jeon M Hay N Expanding the concepts of cancer metabolism. Exp Mol Med. (2018) 50:32 10.1038/s12276-018-0070-9 29657329 PMC5938029

[25] Lee D-K Na E Park S Park JH Lim J Kwon SW. *In Vitro* tracking of intracellular metabolism-derived cancer volatiles via isotope labeling. ACS Central Sci. (2018) 4:1037–44. 10.1021/acscentsci.8b0029 30159401 PMC6107874

[26] Lee D-K Long NP Jung J Kim TJ Na E Kang YP. Integrative lipidomic and transcriptomic analysis of X-linked adrenoleukodystrophy reveals distinct lipidome signatures between adrenomyeloneuropathy and childhood cerebral adrenoleukodystrophy. Biochem Biophys Res Commu. (2018) 508:563–9. 10.1016/j.bbrc.2018.11.123 30509496

[27] Giudetti AM De Domenico S Ragusa A Lunetti P Gaballo A Franck J. A specific lipid metabolic profile is associated with the epithelial mesenchymal transition program. Biochim Biophys Acta Mol Cell Biol Lipids. (2019) 1864:344–57. 10.1016/j.bbalip.2018.12.011 30578966

[28] Dongre A Weinberg RA. New insights into the mechanisms of epithelial–mesenchymal transition and implications for cancer. Nat Rev Mol Cell Biol. (2018) 20:69–84. 10.1038/s41580-018-0080-4 30459476

[29] Kim YJ Koo G-B Lee J-Y Moon H-S Kim D-G Lee D-G. A microchip filter device incorporating slit arrays and 3-D flow for detection of circulating tumor cells using CAV1-EpCAM conjugated microbeads. Biomaterials. (2014) 35:7501–10. 10.1016/j.biomaterials.2014.05.039 24917030

[30] Lee WJ Kim SC Yoon J-H Yoon SJ Lim J Kim Y-S. Meta-analysis of tumor stem-like breast cancer cells using gene set and network analysis. PloS ONE. (2016) 11:e0148818. 10.1371/journal.pone.0148818 26870956 PMC4752453

[31] Kim YS Morgan MJ Choksi S Liu ZG. TNF-induced activation of the Nox1 NADPH oxidase and its role in the induction of necrotic cell death. Mol Cell. (2007) 26:675–87. 10.1016/j.molcel.2007.04.021 17560373

[32] Kang YP Lee WJ Hong JY Lee SB Park JH Kim D. Novel approach for analysis of bronchoalveolar lavage fluid (BALF) using HPLC-QTOF-MS-based lipidomics: lipid levels in asthmatics and corticosteroid-treated asthmatic patients. J Proteome Res. (2014) 13:3919–29. 10.1021/pr5002059 25040188

[33] Yi L He J Liang Y Yuan D Gao H Zhou H. Simultaneously quantitative measurement of comprehensive profiles of esterified and non-esterified fatty acid in plasma of type 2 diabetic patients. Chem Phys Lipids. (2007) 150:204–16. 10.1016/j.chemphyslip.2007.08.002 17880934

[34] Chong J Soufan O Li C Caraus I Li S Bourque G. MetaboAnalyst 4.0: towards more transparent and integrative metabolomics analysis. Nucleic Acids Res. (2018) 46:W486–94. 10.1093/nar/gky310 29762782 PMC6030889

[35] Subramanian A Tamayo P Mootha VK Mukherjee S Ebert BL Gillette MA. Gene set enrichment analysis: a knowledge-based approach for interpreting genome-wide expression profiles. Proc Natl Acad Sci. (2005) 102:15545–50. 10.1073/pnas.0506580102 16199517 PMC1239896

[36] Jézéquel P Campone M Gouraud W Guérin-Charbonnel C Leux C Ricolleau G. bc-GenExMiner: an easy-to-use online platform for gene prognostic analyses in breast cancer. Breast Cancer Res Treat. (2012) 131:765–75. 10.1007/s10549-011-1457-7 21452023

[37] Jézéquel P Frénel J-S Campion L Guérin-Charbonnel C Gouraud W Ricolleau G. bc-GenExMiner 3.0: new mining module computes breast cancer gene expression correlation analyses. Database. (2013) 2013:bas060. 10.1093/database/bas060 23325629 PMC3548333

[38] Tang Z Li C Kang B Gao G Li C Zhang Z. GEPIA: a web server for cancer and normal gene expression profiling and interactive analyses. Nucleic Acids Res. (2017) 45:W98–102. 10.1093/nar/gkx247 28407145 PMC5570223

[39] Pauter AM Olsson P Asadi A Herslöf B Csikasz RI Zadravec D. ELOVL2 ablation demonstrates that systemic DHA is endogenously produced and is essential for lipid homeostasis in mice. J Lipid Res. (2014) 55:718–28. 10.1194/jlr.M046151 24489111 PMC3966705

[40] Botolin D Wang Y Christian B Jump DB. Docosahexaneoic acid (22: 6, n-3) regulates rat hepatocyte SREBP-1 nuclear abundance by Erk-and 26S proteasome-dependent pathways. J Lipid Res. (2006) 47:181–92. 10.1194/jlr.M500365-JLR200 16222032 PMC2764363

[41] Hannah VC Ou J Luong A Goldstein JL Brown MS. Unsaturated fatty acids down-regulate srebp isoforms 1a and 1c by two mechanisms in HEK-293 cells. J Biol Chem. (2001) 276:4365–72. 10.1074/jbc.M007273200 11085986

[42] Swinnen JV Heemers H Deboel L Foufelle F Heyns W Verhoeven G. Stimulation of tumor-associated fatty acid synthase expression by growth factor activation of the sterol regulatory element-binding protein pathway. Oncogene. (2000) 19:5173–81. 10.1038/sj.onc.1203889 11064454

[43] Baker S Ali I Silins I Pyysalo S Guo Y Högberg J. Cancer Hallmarks Analytics Tool (CHAT): a text mining approach to organize and evaluate scientific literature on cancer. Bioinformatics. (2017) 33:3973–3981. 10.1093/bioinformatics/btx454 29036271 PMC5860084

[44] Neve RM Chin K Fridlyand J Yeh J Baehner FL Fevr T. A collection of breast cancer cell lines for the study of functionally distinct cancer subtypes. Cancer Cell. (2006) 10:515–27. 10.1016/j.ccr.2006.10.008 17157791 PMC2730521

[45] Sarrió D Rodriguez-Pinilla SM Hardisson D Cano A Moreno-Bueno G Palacios J. Epithelial-mesenchymal transition in breast cancer relates to the basal-like phenotype. Cancer Res. (2008) 68:989–97. 10.1158/0008-5472.CAN-07-2017 18281472

[46] Gonzalez-Bengtsson A Asadi A Gao H Dahlman-Wright K Jacobsson A. Estrogen enhances the expression of the polyunsaturated fatty acid elongase ELOVL2 via eralpha in breast cancer cells. PLoS ONE. (2016) 11:e0164241. 10.1371/journal.pone.0164241 27788154 PMC5082882

[47] Vasaikar SV Straub P Wang J Zhang B. LinkedOmics: analyzing multi-omics data within and across 32 cancer types. Nucleic Acids Res. (2017) 46:D956–63. 10.1093/nar/gkx1090 29136207 PMC5753188

[48] Long NP Jung KH Anh NH Yan HH Nghi TD Park S. An integrative data mining and omics-based translational model for the identification and validation of oncogenic biomarkers of pancreatic cancer. Cancers. (2019) 11:e155. 10.3390/cancers11020155 30700038 PMC6407035

[49] Yang K Han X. Lipidomics: techniques, applications, and outcomes related to biomedical sciences. Trends Biochem Sci. (2016) 41:954–69. 10.1016/j.tibs.2016.08.010 27663237 PMC5085849

[50] Kang YP Ward NP DeNicola GM. Recent advances in cancer metabolism: a technological perspective. Exp Mol Med. (2018) 50:31. 10.1038/s12276-018-0027-z 29657324 PMC5938018

[51] Maan M Peters JM Dutta M Patterson AD. Lipid metabolism and lipophagy in cancer. Biochem Biophys Res Commu. (2018) 504:582–9. 10.1016/j.bbrc.2018.02.097 29438712 PMC6086774

[52] Braig S. Chemical genetics in tumor lipogenesis. Biotechnol Adv. (2018) 36:1724–9. 10.1016/j.biotechadv.2018.02.007 29447918

[53] Zalba S ten Hagen TL. Cell membrane modulation as adjuvant in cancer therapy. Cancer Treat Rev. (2017) 52:48–57. 10.1016/j.ctrv.2016.10.008 27889637 PMC5195909

[54] Patra SK. Dissecting lipid raft facilitated cell signaling pathways in cancer. Biochim Biophys Acta Rev Cancer. (2008) 1785:182–206. 10.1016/j.bbcan.2007.11.002 18166162

[55] Chajès V Thiébaut AC Rotival M Gauthier E Maillard V Boutron-Ruault M-C. Association between serum trans-monounsaturated fatty acids and breast cancer risk in the E3N-EPIC, Study. Am J Epidemiol. (2008) 167:1312–20. 10.1093/aje/kwn069 18390841 PMC2679982

[56] Fritz V Benfodda Z Rodier G Henriquet C Iborra F Avances C. Abrogation of de novo lipogenesis by stearoyl-CoA desaturase 1 inhibition interferes with oncogenic signaling and blocks prostate cancer progression in mice. Mol Cancer Ther. (2010) 9:1740–54. 10.1158/1535-7163.MCT-09-1064 20530718 PMC3315476

[57] Deng X Dong Q Bridges D Raghow R Park EA Elam MB. Docosahexaenoic acid inhibits proteolytic processing of sterol regulatory element-binding protein-1c (SREBP-1c) via activation of AMP-activated kinase. Biochim Biophys Acta Mol Cell Biol Lipids. (2015) 1851:1521–9. 10.1016/j.bbalip.2015.08.007 26327595

[58] Alvaro A Rosales R Masana L Vallvé J-C. Polyunsaturated fatty acids down-regulate *in vitro* expression of the key intestinal cholesterol absorption protein NPC1L1: no effect of monounsaturated nor saturated fatty acids. J Nutr Biochem. (2010) 21:518–25. 10.1016/j.jnutbio.2009.02.010 19443194

[59] Furuta E Pai SK Zhan R Bandyopadhyay S Watabe M Mo Y-Y. Fatty acid synthase gene is up-regulated by hypoxia via activation of Akt and sterol regulatory element binding protein-1. Cancer Res. (2008) 68:1003–11. 10.1158/0008-5472.CAN-07-2489 18281474

[60] Kamphorst JJ Cross JR Fan J de Stanchina E Mathew R White EP. Hypoxic and ras-transformed cells support growth by scavenging unsaturated fatty acids from lysophospholipids. Proc Natl Acad Sci. (2013) 110:8882–7. 10.1073/pnas.1307237110 23671091 PMC3670379

[61] Bellini L Campana M Rouch C Chacinska M Bugliani M Meneyrol K. Protective role of the ELOVL2/docosahexaenoic acid axis in glucolipotoxicity-induced apoptosis in rodent beta cells and human islets. Diabetologia. (2018) 61:1780–93. 10.1007/s00125-018-4629-8 29754287

[62] Cruciani-Guglielmacci C Bellini L Denom J Oshima M Fernandez N Normandie-Levi P. Molecular phenotyping of multiple mouse strains under metabolic challenge uncovers a role for Elovl2 in glucose-induced insulin secretion. Mol Metab. (2017) 6:340–51. 10.1016/j.molmet.2017.01.009 28377873 PMC5369210

[63] Long NP Jung KH Yoon SJ Anh NH Nghi TD Kang YP. Systematic assessment of cervical cancer initiation and progression uncovers genetic panels for deep learning-based early diagnosis and proposes novel diagnostic and prognostic biomarkers. Oncotarget. (2017) 8:109436–56. 10.18632/oncotarget.22689 29312619 PMC5752532

