# Supplementary material for: Spheroid-Induced Epithelial-Mesenchymal Transition Provokes Global Alterations of Breast Cancer Lipidome: A Multi-Layered Omics Analysis
Source: Front Oncol. 2019 Mar 21;9:145. doi: 10.3389/fonc.2019.00145 (PMC6437068; doi:10.3389/fonc.2019.00145)
Supplement: Supplementary file 1 [file Data_Sheet_1.pdf]

Supplementary Material

1 Supplementary Figures and Tables

1.1 Supplementary Figures

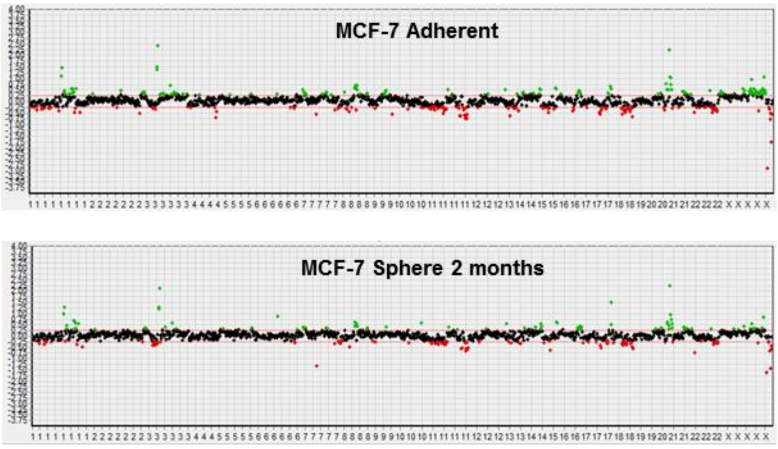

Supplementary Figure 1. Comparative genomic hybridization (CGH) analysis between Adherent and Sphere.

Supplementary Figure 2. Internal spectral library and lipid identification process.

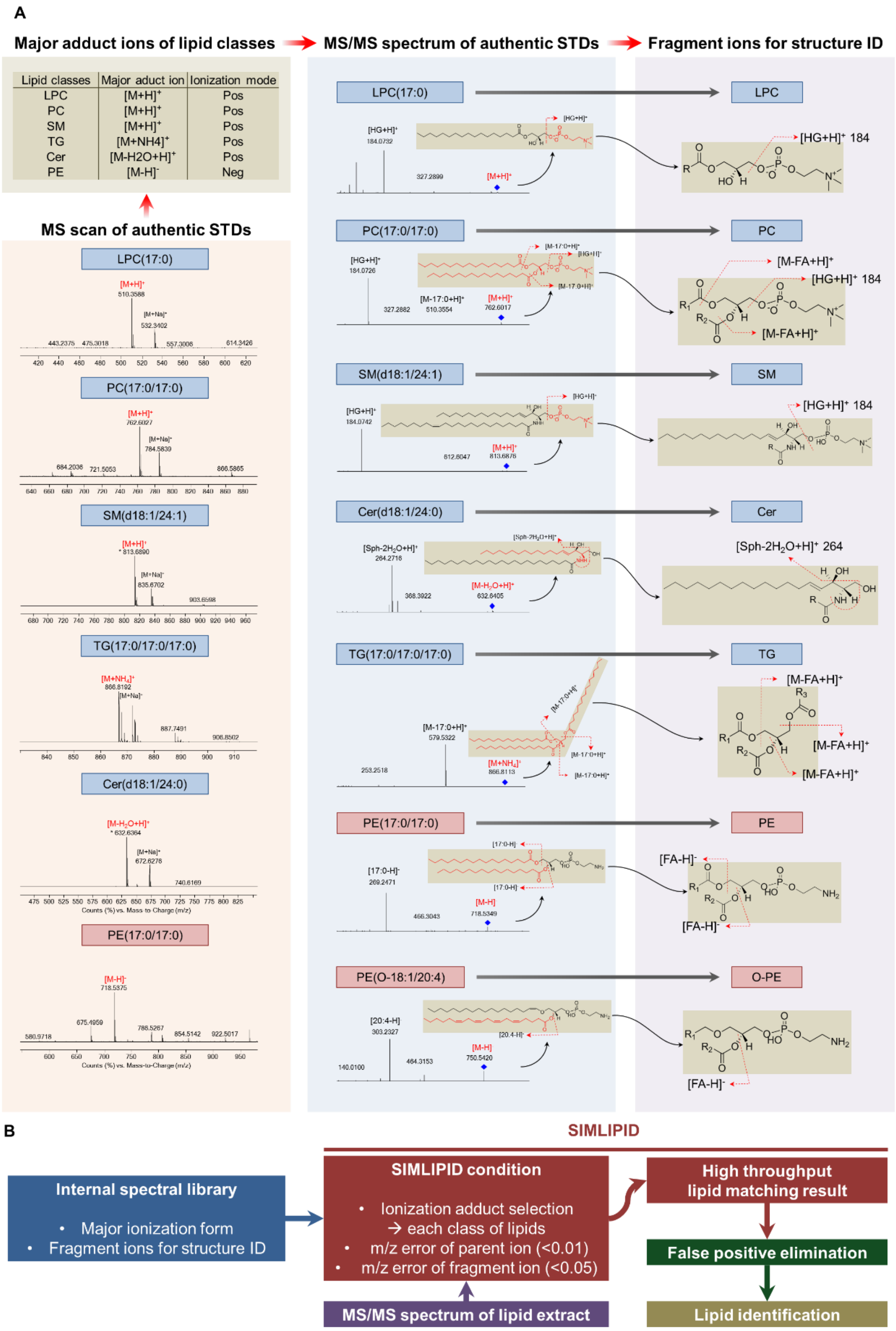

Supplementary Figure 3. MS and MS/MS spectrum based identification of LPC, PC, O-PC, SM, TG, Cer, PE, and O-PE.

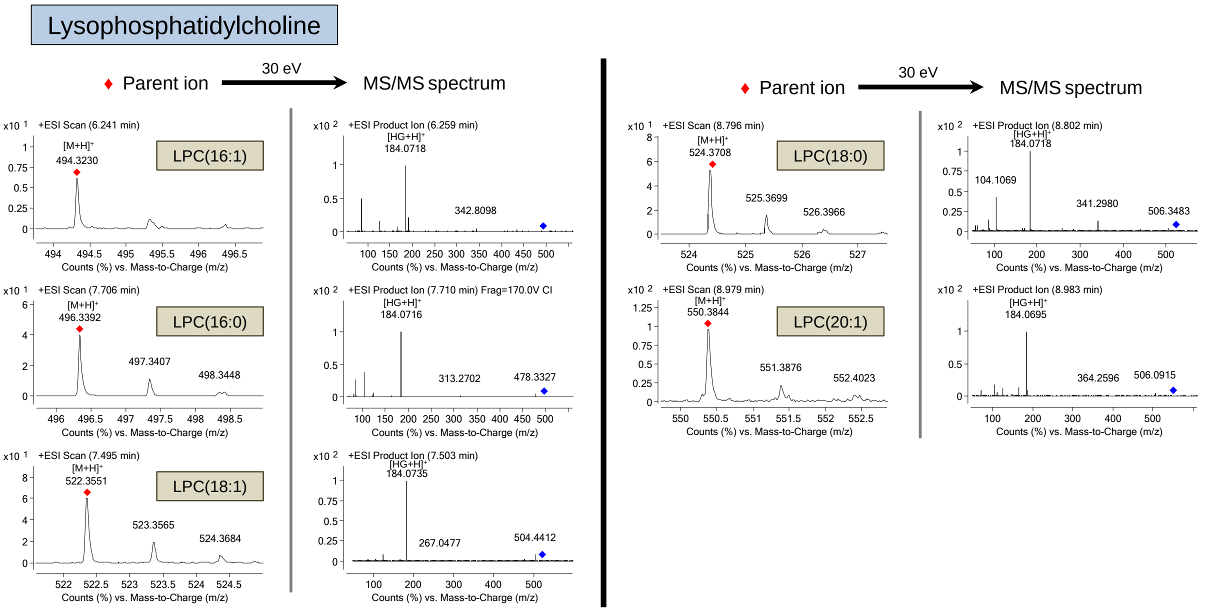

Continued

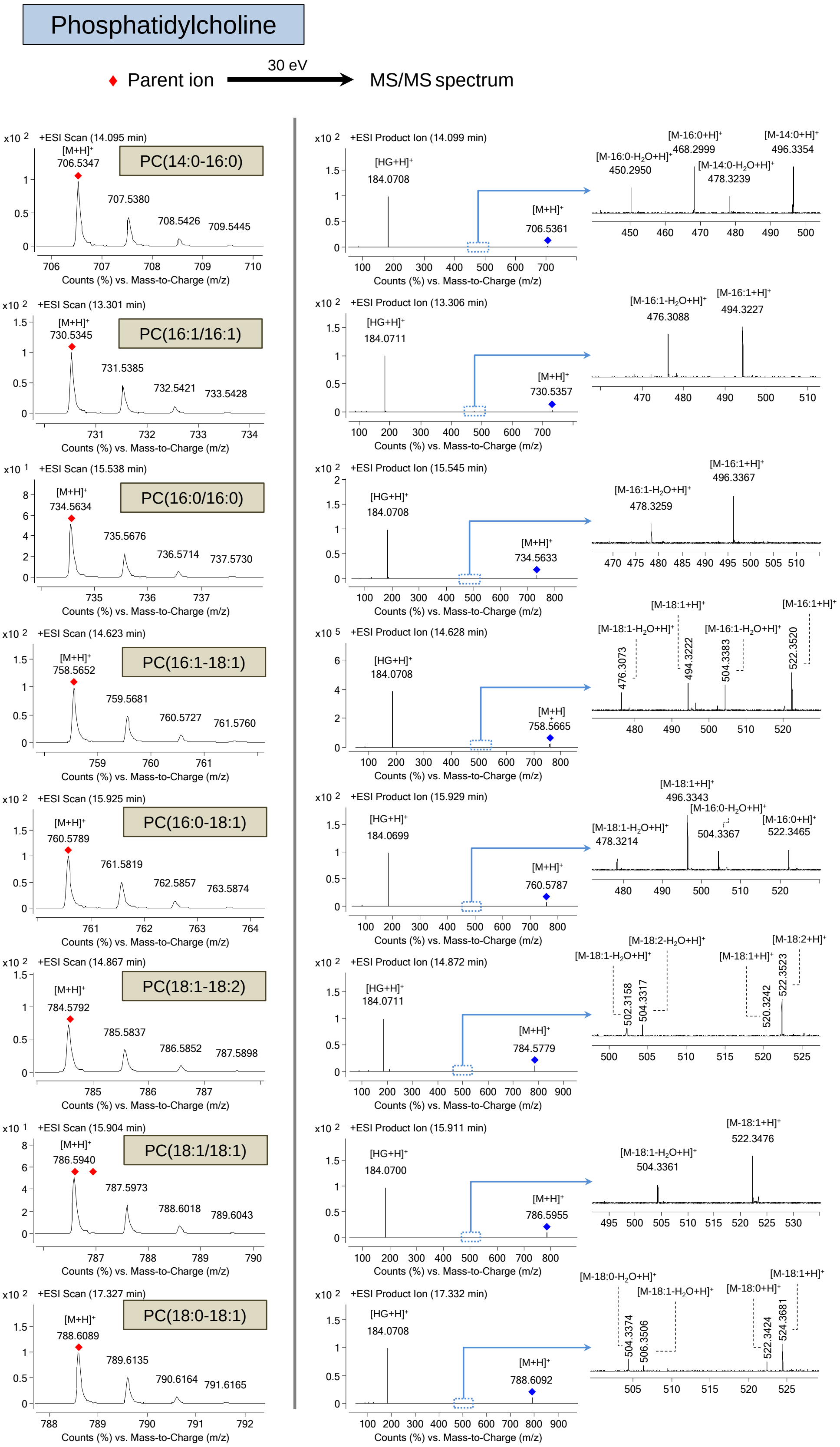
Phosphatidylcholine
Parent ion
30 eV
MS/MS spectrum
x10 2 +ESI Scan (14.095 min)
[M+H]+ 706.5347
PC(14:0-16:0)
707.5380
708.5426
709.5445
Counts (%) vs. Mass-to-Charge (m/z)
x10 2 +ESI Product Ion (14.099 min)
[HG+H]+ 184.0708
[M+H]+ 706.5361
[M-16:0+H]+ 468.2999
[M-14:0+H]+ 496.3354
[M-16:0-H2O+H]+ 450.2950
[M-14:0-H2O+H]+ 478.3239
x10 2 +ESI Scan (13.301 min)
[M+H]+ 730.5345
PC(16:1/16:1)
731.5385
732.5421
733.5428
x10 2 +ESI Product Ion (13.306 min)
[HG+H]+ 184.0711
[M+H]+ 730.5357
[M-16:1-H2O+H]+ 476.3088
[M-16:1+H]+ 494.3227
x10 1 +ESI Scan (15.538 min)
[M+H]+ 734.5634
PC(16:0/16:0)
735.5676
736.5714
737.5730
x10 2 +ESI Product Ion (15.545 min)
[HG+H]+ 184.0708
[M+H]+ 734.5633
[M-16:1-H2O+H]+ 478.3259
[M-16:1+H]+ 496.3367
x10 2 +ESI Scan (14.623 min)
[M+H]+ 758.5652
PC(16:1-18:1)
759.5681
760.5727
761.5760
x10 5 +ESI Product Ion (14.628 min)
[HG+H]+ 184.0708
[M+H] 758.5665
[M-18:1-H2O+H]+ 476.3073
[M-18:1+H]+ 494.3222
[M-16:1-H2O+H]+ 504.3383
[M-16:1+H]+ 522.3520
x10 2 +ESI Scan (15.925 min)
[M+H]+ 760.5789
PC(16:0-18:1)
761.5819
762.5857
763.5874
x10 2 +ESI Product Ion (15.929 min)
[HG+H]+ 184.0699
[M+H]+ 760.5787
[M-18:1+H]+ 496.3343
[M-16:0-H2O+H]+ 504.3367
[M-18:1-H2O+H]+ 478.3214
[M-16:0+H]+ 522.3465
x10 2 +ESI Scan (14.867 min)
[M+H]+ 784.5792
PC(18:1-18:2)
785.5837
786.5852
787.5898
x10 2 +ESI Product Ion (14.872 min)
[HG+H]+ 184.0711
[M+H]+ 784.5779
[M-18:2-H2O+H]+
[M-18:1-H2O+H]+
[M-18:2+H]+
[M-18:1+H]+
502.3158
504.3317
520.3242
522.3523
x10 1 +ESI Scan (15.904 min)
[M+H]+ 786.5940
PC(18:1/18:1)
787.5973
788.6018
789.6043
x10 2 +ESI Product Ion (15.911 min)
[HG+H]+ 184.0700
[M+H]+ 786.5955
[M-18:1-H2O+H]+ 504.3361
[M-18:1+H]+ 522.3476
x10 2 +ESI Scan (17.327 min)
[M+H]+ 788.6089
PC(18:0-18:1)
789.6135
790.6164
791.6165
x10 2 +ESI Product Ion (17.332 min)
[HG+H]+ 184.0708
[M+H]+ 788.6092
[M-18:0-H2O+H]+
[M-18:1-H2O+H]+
[M-18:0+H]+
[M-18:1+H]+
504.3374
506.3506
522.3424
524.3681

Continued

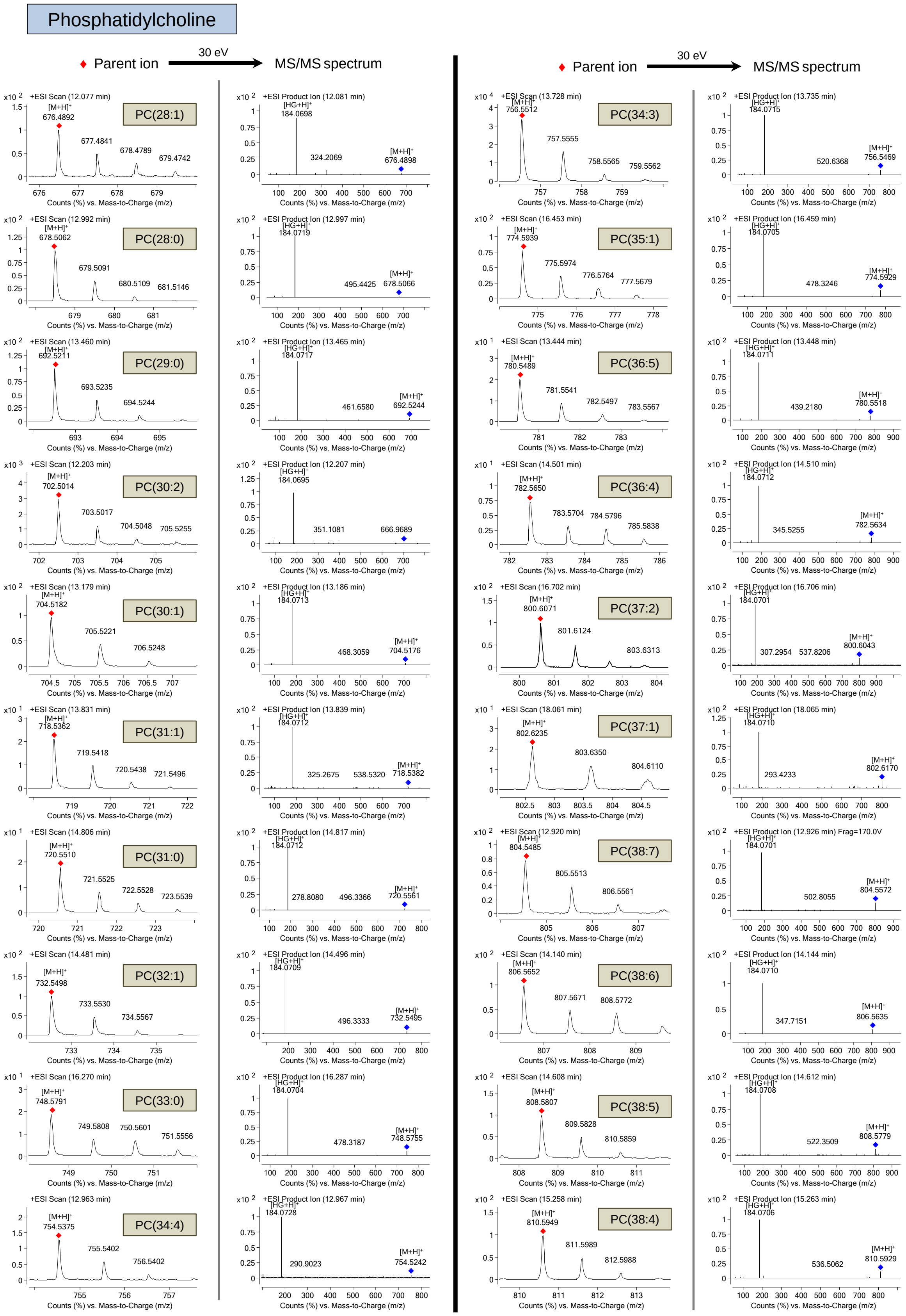
Phosphatidylcholine
Parent ion
30 eV
MS/MS spectrum
Parent ion
30 eV
MS/MS spectrum
PC(28:1)
PC(28:0)
PC(29:0)
PC(30:2)
PC(30:1)
PC(31:1)
PC(31:0)
PC(32:1)
PC(33:0)
PC(34:4)
PC(34:3)
PC(35:1)
PC(36:5)
PC(36:4)
PC(37:2)
PC(37:1)
PC(38:7)
PC(38:6)
PC(38:5)
PC(38:4)

Continued

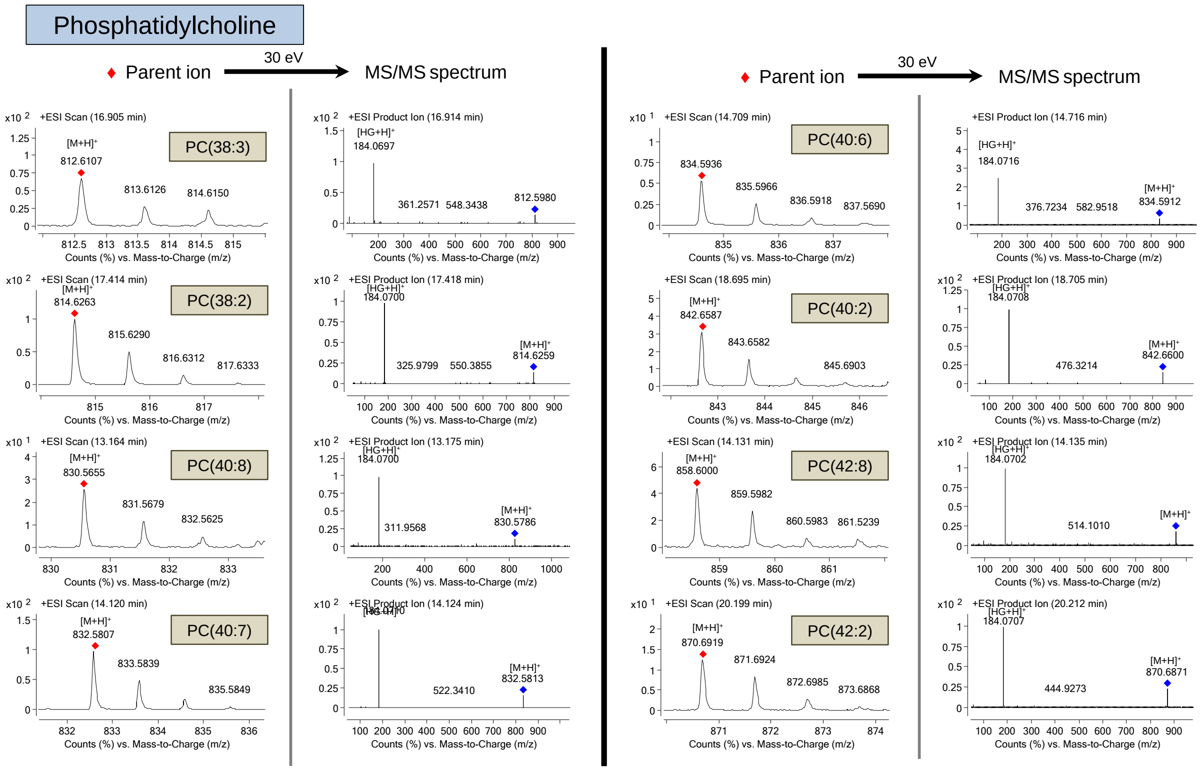
Phosphatidylcholine
Parent ion
30 eV
MS/MS spectrum
PC(38:3)
PC(38:2)
PC(40:8)
PC(40:7)
PC(40:6)
PC(40:2)
PC(42:8)
PC(42:2)
Counts (%) vs. Mass-to-Charge (m/z)

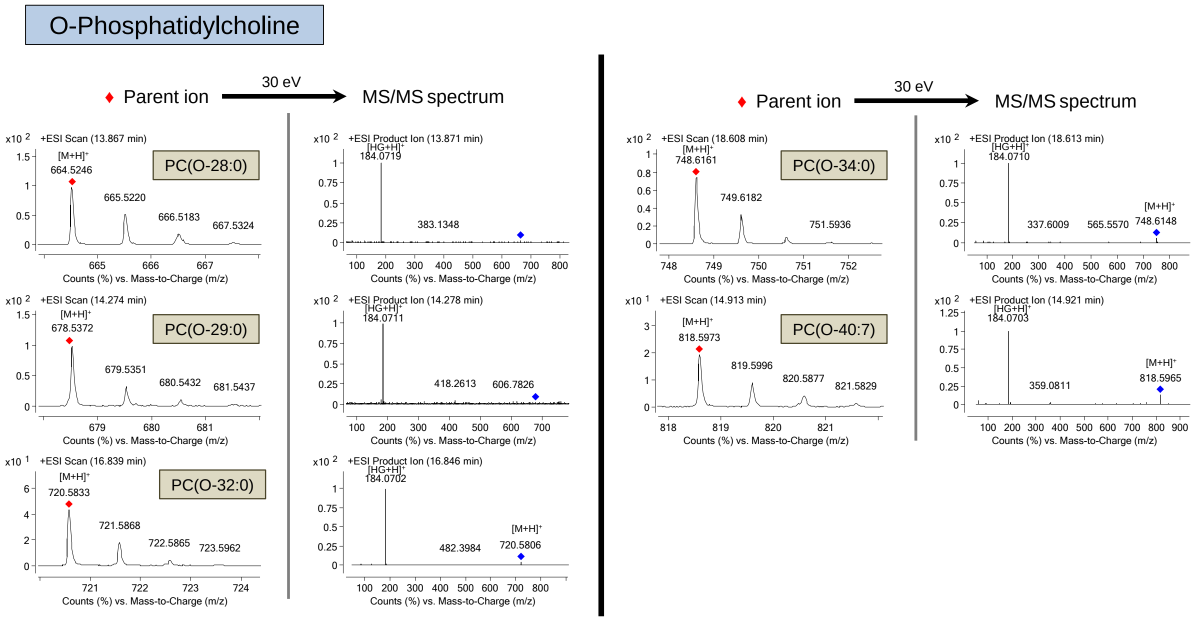
O-Phosphatidylcholine
Parent ion
30 eV
MS/MS spectrum
PC(O-28:0)
PC(O-29:0)
PC(O-32:0)
PC(O-34:0)
PC(O-40:7)
Counts (%) vs. Mass-to-Charge (m/z)

Continued

Sphingomyelin

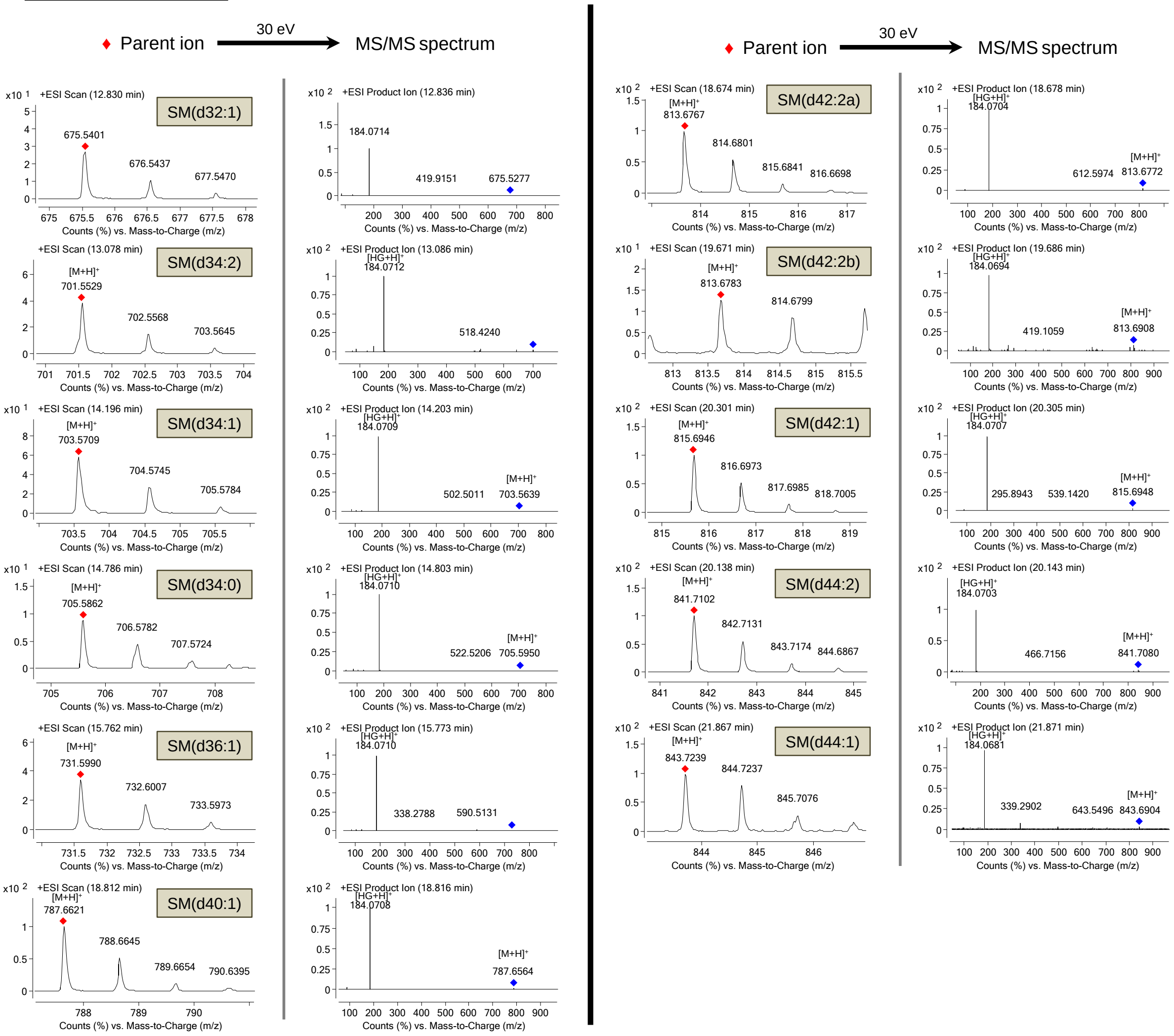

Continued

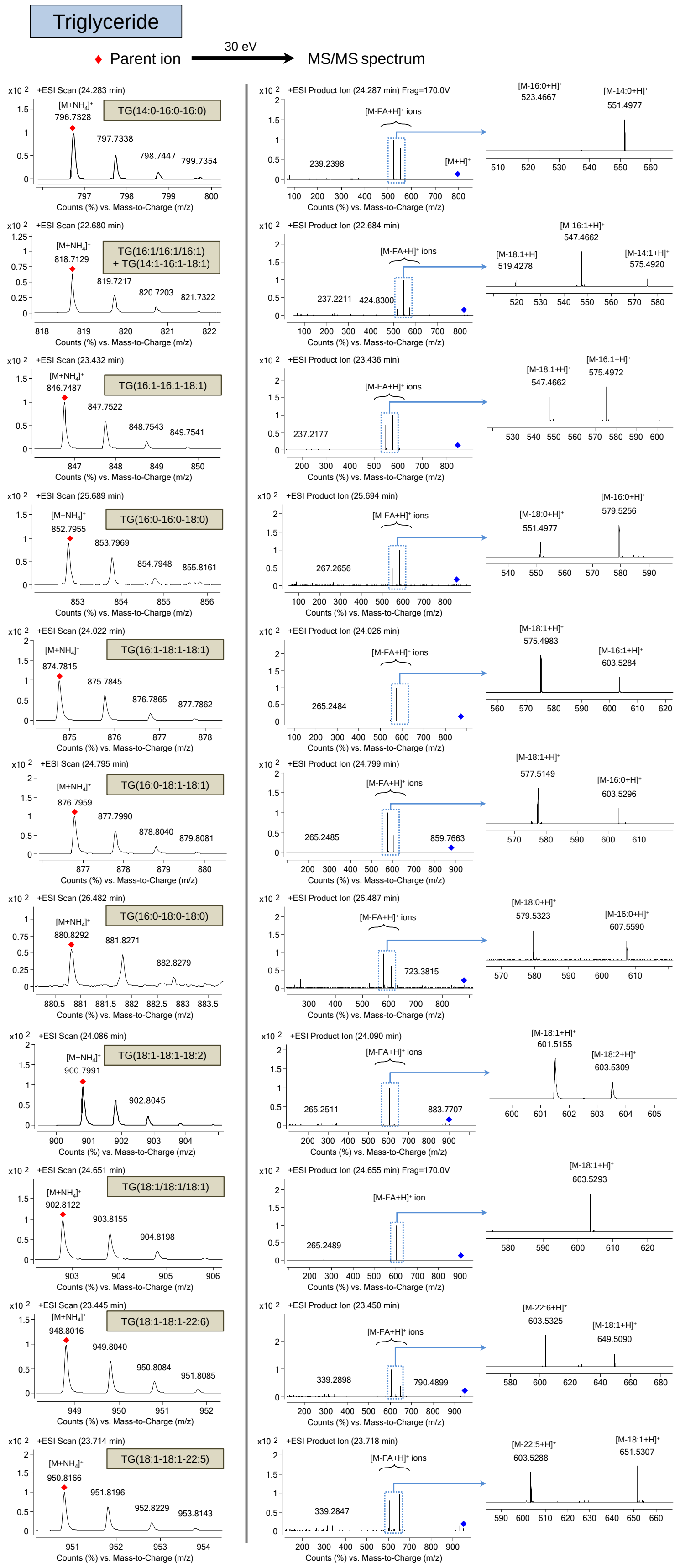
Triglyceride
Parent ion
30 eV
MS/MS spectrum
TG(14:0-16:0-16:0)
TG(16:1/16:1/16:1) + TG(14:1-16:1-18:1)
TG(16:1-16:1-18:1)
TG(16:0-16:0-18:0)
TG(16:1-18:1-18:1)
TG(16:0-18:1-18:1)
TG(16:0-18:0-18:0)
TG(18:1-18:1-18:2)
TG(18:1/18:1/18:1)
TG(18:1-18:1-22:6)
TG(18:1-18:1-22:5)
Counts (%) vs. Mass-to-Charge (m/z)

Continued

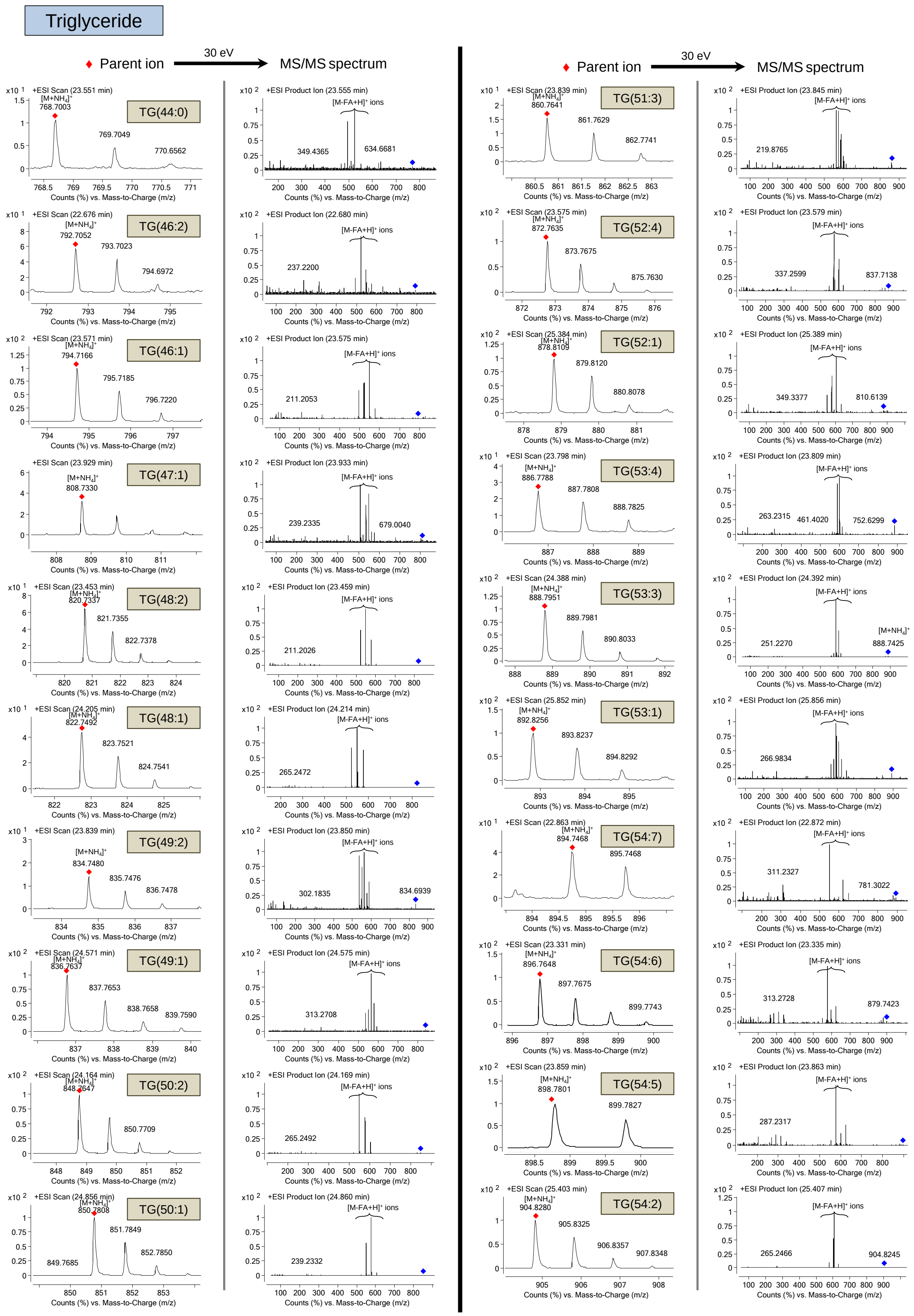
Triglyceride
Parent ion
30 eV
MS/MS spectrum
TG(44:0)
768.7003
769.7049
770.6562
349.4365
634.6681
TG(46:2)
792.7052
793.7023
794.6972
237.2200
TG(46:1)
794.7166
795.7185
796.7220
211.2053
TG(47:1)
808.7330
239.2335
679.0040
TG(48:2)
820.7337
821.7355
822.7378
211.2026
TG(48:1)
822.7492
823.7521
824.7541
265.2472
TG(49:2)
834.7480
835.7476
836.7478
302.1835
834.6939
TG(49:1)
836.7637
837.7653
838.7658
839.7590
313.2708
TG(50:2)
848.7647
850.7709
265.2492
TG(50:1)
850.7808
851.7849
849.7685
852.7850
239.2332
TG(51:3)
860.7641
861.7629
862.7741
219.8765
TG(52:4)
872.7635
873.7675
875.7630
337.2599
837.7138
TG(52:1)
878.8109
879.8120
880.8078
349.3377
810.6139
TG(53:4)
886.7788
887.7808
888.7825
263.2315
461.4020
752.6299
TG(53:3)
888.7951
889.7981
890.8033
251.2270
888.7425
TG(53:1)
892.8256
893.8237
894.8292
266.9834
TG(54:7)
894.7468
895.7468
311.2327
781.3022
TG(54:6)
896.7648
897.7675
899.7743
313.2728
879.7423
TG(54:5)
898.7801
899.7827
287.2317
TG(54:2)
904.8280
905.8325
906.8357
907.8348
265.2466
904.8245
[M+NH4]+
[M-FA+H]+ ions
Counts (%) vs. Mass-to-Charge (m/z)

Continued

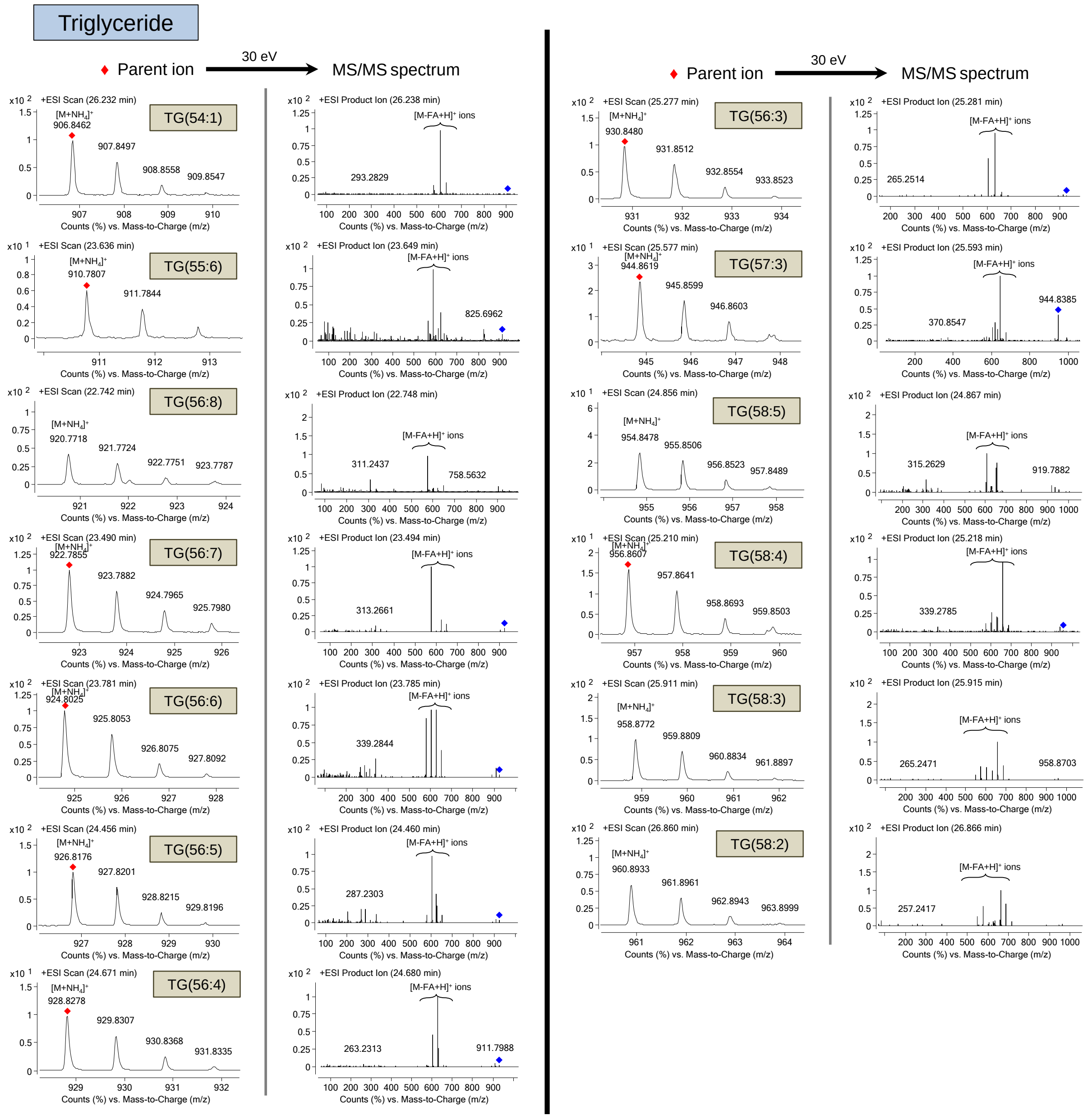
Triglyceride
Parent ion
30 eV
MS/MS spectrum
TG(54:1)
[M+NH4]+ 906.8462
907.8497
908.8558
909.8547
[M-FA+H]+ ions
293.2829
TG(55:6)
[M+NH4]+ 910.7807
911.7844
825.6962
TG(56:8)
[M+NH4]+ 920.7718
921.7724
922.7751
923.7787
311.2437
758.5632
TG(56:7)
[M+NH4]+ 922.7855
923.7882
924.7965
925.7980
313.2661
TG(56:6)
[M+NH4]+ 924.8025
925.8053
926.8075
927.8092
339.2844
TG(56:5)
[M+NH4]+ 926.8176
927.8201
928.8215
929.8196
287.2303
TG(56:4)
[M+NH4]+ 928.8278
929.8307
930.8368
931.8335
263.2313
911.7988
TG(56:3)
[M+NH4]+ 930.8480
931.8512
932.8554
933.8523
265.2514
TG(57:3)
[M+NH4]+ 944.8619
945.8599
946.8603
370.8547
944.8385
TG(58:5)
[M+NH4]+ 954.8478
955.8506
956.8523
957.8489
315.2629
919.7882
TG(58:4)
[M+NH4]+ 956.8607
957.8641
958.8693
959.8503
339.2785
TG(58:3)
[M+NH4]+ 958.8772
959.8809
960.8834
961.8897
265.2471
958.8703
TG(58:2)
[M+NH4]+ 960.8933
961.8961
962.8943
963.8999
257.2417
Counts (%) vs. Mass-to-Charge (m/z)

Continued

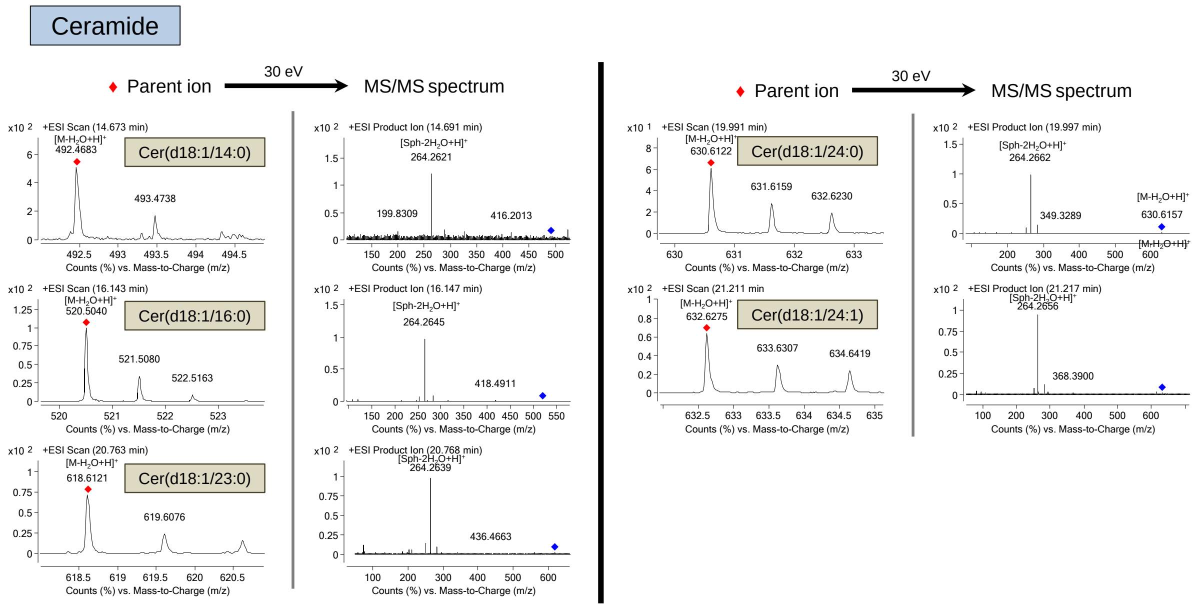
Ceramide
Parent ion
30 eV
MS/MS spectrum
+ESI Scan (14.673 min)
[M-H2O+H]+
492.4683
Cer(d18:1/14:0)
493.4738
Counts (%) vs. Mass-to-Charge (m/z)
+ESI Product Ion (14.691 min)
[Sph-2H2O+H]+
264.2621
199.8309
416.2013
+ESI Scan (16.143 min)
520.5040
Cer(d18:1/16:0)
521.5080
522.5163
+ESI Product Ion (16.147 min)
264.2645
418.4911
+ESI Scan (20.763 min)
618.6121
Cer(d18:1/23:0)
619.6076
+ESI Product Ion (20.768 min)
264.2639
436.4663
+ESI Scan (19.991 min)
630.6122
Cer(d18:1/24:0)
631.6159
632.6230
+ESI Product Ion (19.997 min)
264.2662
349.3289
630.6157
+ESI Scan (21.211 min
632.6275
Cer(d18:1/24:1)
633.6307
634.6419
+ESI Product Ion (21.217 min)
264.2656
368.3900

Continued

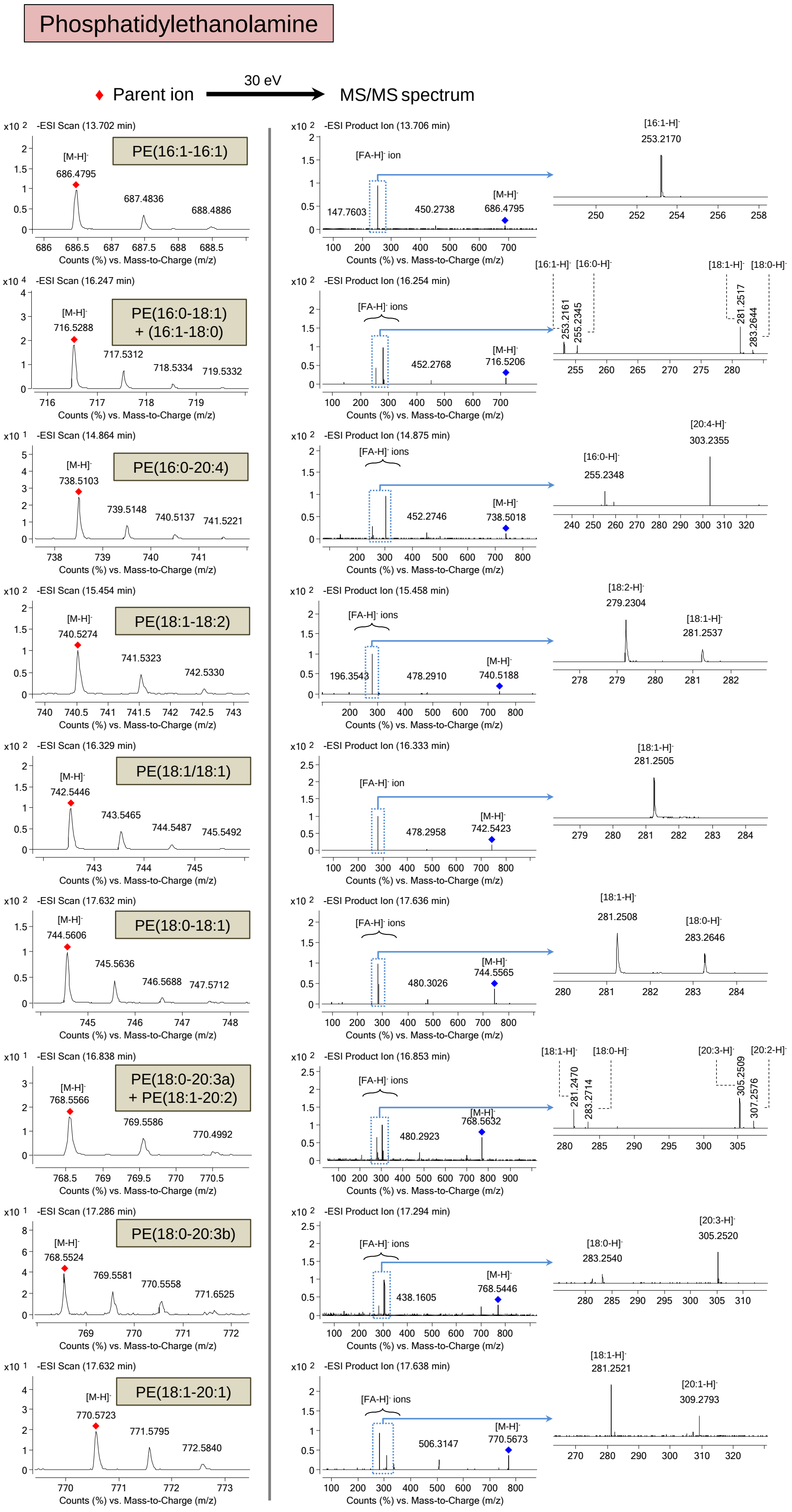

Phosphatidylethanolamine
Parent ion
30 eV
MS/MS spectrum
PE(16:1-16:1)
PE(16:0-18:1) + (16:1-18:0)
PE(16:0-20:4)
PE(18:1-18:2)
PE(18:1/18:1)
PE(18:0-18:1)
PE(18:0-20:3a) + PE(18:1-20:2)
PE(18:0-20:3b)
PE(18:1-20:1)
-ESI Scan (13.702 min)
-ESI Product Ion (13.706 min)
-ESI Scan (16.247 min)
-ESI Product Ion (16.254 min)
-ESI Scan (14.864 min)
-ESI Product Ion (14.875 min)
-ESI Scan (15.454 min)
-ESI Product Ion (15.458 min)
-ESI Scan (16.329 min)
-ESI Product Ion (16.333 min)
-ESI Scan (17.632 min)
-ESI Product Ion (17.636 min)
-ESI Scan (16.838 min)
-ESI Product Ion (16.853 min)
-ESI Scan (17.286 min)
-ESI Product Ion (17.294 min)
-ESI Scan (17.632 min)
-ESI Product Ion (17.638 min)
Counts (%) vs. Mass-to-Charge (m/z)
[M-H]-
[FA-H]- ion
[FA-H]- ions

Continued

frontiers

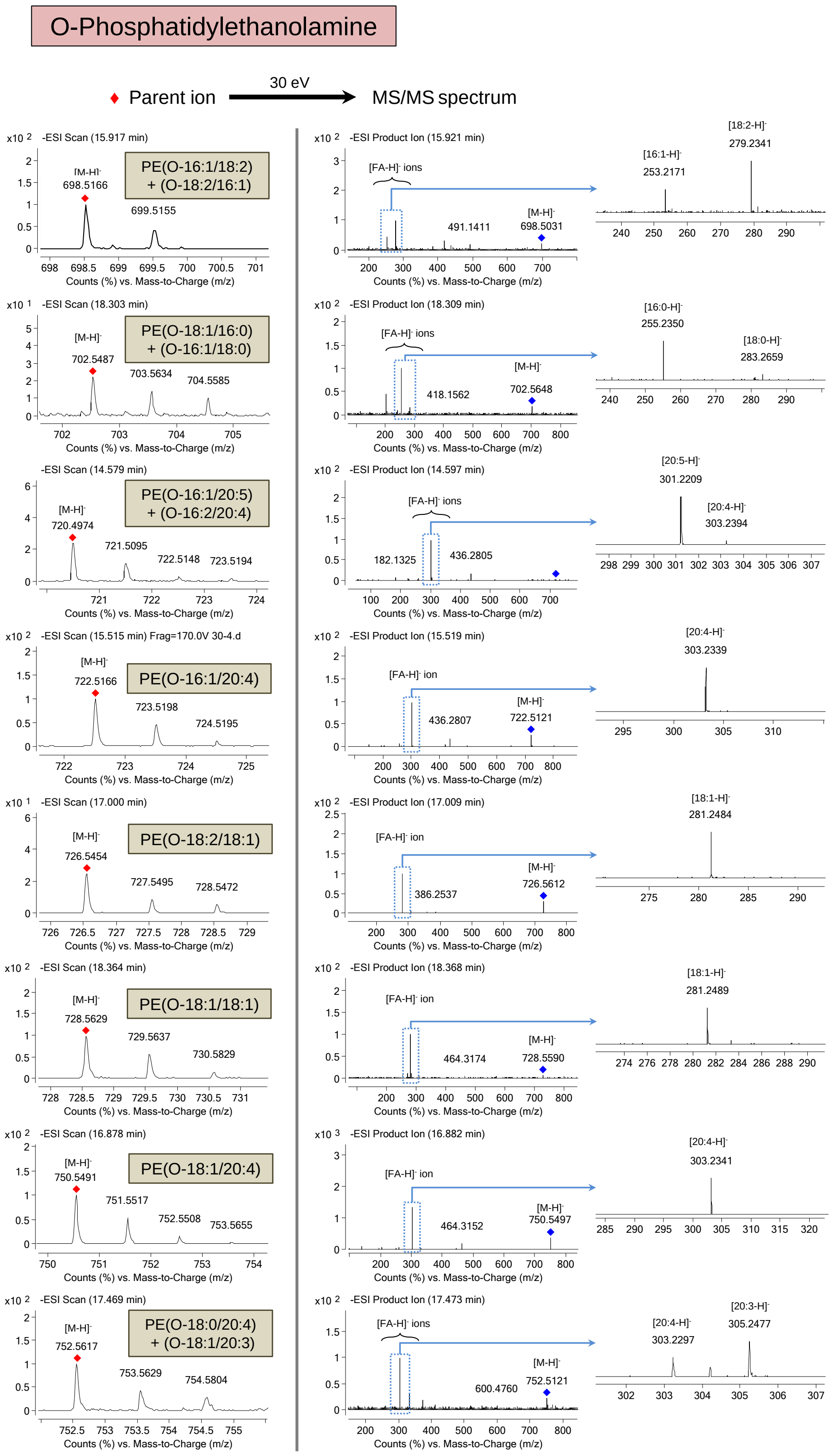
O-Phosphatidylethanolamine
Parent ion
30 eV
MS/MS spectrum
-ESI Scan (15.917 min)
PE(O-16:1/18:2) + (O-18:2/16:1)
[M-H]- 698.5166
699.5155
-ESI Product Ion (15.921 min)
[FA-H]- ions
491.1411
[M-H]- 698.5031
[16:1-H]- 253.2171
[18:2-H]- 279.2341
-ESI Scan (18.303 min)
PE(O-18:1/16:0) + (O-16:1/18:0)
[M-H]- 702.5487
703.5634
704.5585
-ESI Product Ion (18.309 min)
[FA-H]- ions
418.1562
[M-H]- 702.5648
[16:0-H]- 255.2350
[18:0-H]- 283.2659
-ESI Scan (14.579 min)
PE(O-16:1/20:5) + (O-16:2/20:4)
[M-H]- 720.4974
721.5095
722.5148
723.5194
-ESI Product Ion (14.597 min)
[FA-H]- ions
182.1325
436.2805
[20:5-H]- 301.2209
[20:4-H]- 303.2394
-ESI Scan (15.515 min) Frag=170.0V 30-4.d
PE(O-16:1/20:4)
[M-H]- 722.5166
723.5198
724.5195
-ESI Product Ion (15.519 min)
[FA-H]- ion
436.2807
[M-H]- 722.5121
[20:4-H]- 303.2339
-ESI Scan (17.000 min)
PE(O-18:2/18:1)
[M-H]- 726.5454
727.5495
728.5472
-ESI Product Ion (17.009 min)
[FA-H]- ion
386.2537
[M-H]- 726.5612
[18:1-H]- 281.2484
-ESI Scan (18.364 min)
PE(O-18:1/18:1)
[M-H]- 728.5629
729.5637
730.5829
-ESI Product Ion (18.368 min)
[FA-H]- ion
464.3174
[M-H]- 728.5590
[18:1-H]- 281.2489
-ESI Scan (16.878 min)
PE(O-18:1/20:4)
[M-H]- 750.5491
751.5517
752.5508
753.5655
-ESI Product Ion (16.882 min)
[FA-H]- ion
464.3152
[M-H]- 750.5497
[20:4-H]- 303.2341
-ESI Scan (17.469 min)
PE(O-18:0/20:4) + (O-18:1/20:3)
[M-H]- 752.5617
753.5629
754.5804
-ESI Product Ion (17.473 min)
[FA-H]- ions
600.4760
[M-H]- 752.5121
[20:4-H]- 303.2297
[20:3-H]- 305.2477
Counts (%) vs. Mass-to-Charge (m/z)

Supplementary Figure 4. PLS-DA scores plot and cross-validation results.

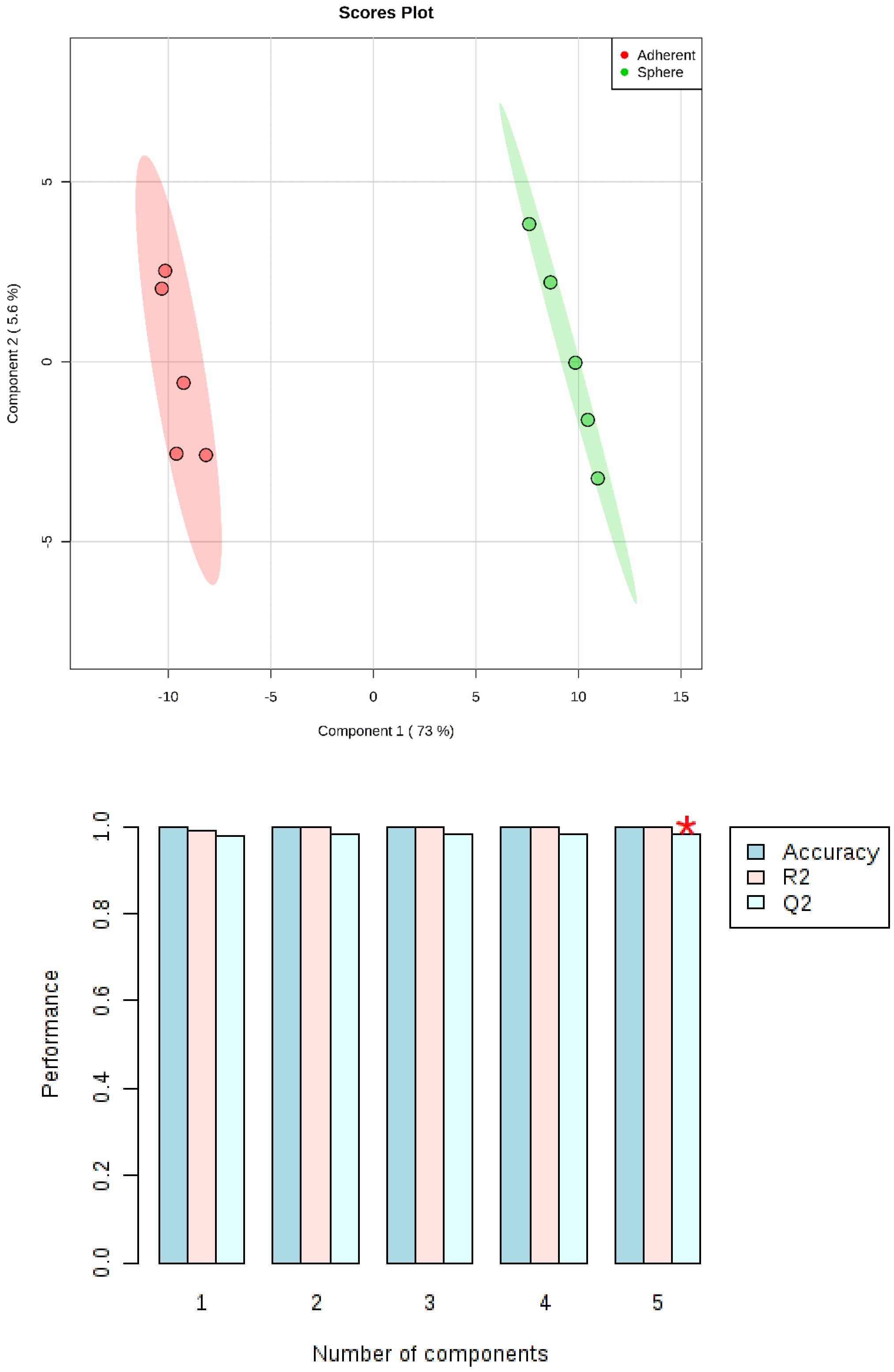

1.2 Supplementary Table

Supplementary Table 1. Relative response factor (RRF) of 12 FAMEs to internal standards.

FAME	Target m/z	RRF (C17:0)	RRF (C23:0)
C16:0	74	1.0	0.9
C16:1	55	0.3	0.3
C18:0	74	1.1	1.0
C18:1	55	0.4	0.4
C18:2	67	0.4	0.4
C20:0	74	1.1	1.0
C20:3n6	79	0.2	0.2
C20:4n6	79	0.3	0.3
C22:0	74	1.1	1.0
C22:5n3	79	0.5	0.4
C22:6n3	79	0.3	0.3
C24:0	74	1.1	1.0

Supplementary Table 2. Compositions of 123 lipid species and 8 lipid classes between adherent and sphere MCF-7 cells

RT (min)	Adduct ion	row m/z	theo m/z	delta m/z	Lipids	Adherent_1	Adherent_2	Adherent_3	Adherent_4	Adherent_5	Sphere_1	Sphere_2	Sphere_3	Sphere_4	Sphere_5	p.value	FDR	log2(Fc)
14.61	[M-H2O+H]+	492.4743	492.4775	0.0032	Cer(d18:1/14:0)	0.00167	0.00194	0.00169	0.00114	0.00110	0.00339	0.00245	0.00591	0.00374	0.00517	0.001	0.003	1.5
16.19	[M-H2O+H]+	520.5038	520.5088	0.0050	Cer(d18:1/16:0)	0.00945	0.01131	0.00815	0.00129	0.00063	0.03684	0.00409	0.06244	0.02655	0.07032	0.032	0.060	2.7
20.90	[M-H2O+H]+	618.6132	618.6183	0.0051	Cer(d18:1/23:0)	0.00536	0.00581	0.00549	0.00285	0.00345	0.00272	0.00190	0.00425	0.00278	0.00400	0.097	0.159	-
21.45	[M-H2O+H]+	632.6293	632.6340	0.0047	Cer(d18:1/24:0)	0.05902	0.06273	0.05691	0.02865	0.03540	0.03919	0.02783	0.06531	0.04240	0.05759	0.855	0.899	-
20.19	[M-H2O+H]+	630.6133	630.6183	0.0050	Cer(d18:1/24:1)	0.16066	0.01247	0.16399	0.08106	0.09863	0.32302	0.22559	0.52945	0.34432	0.44763	0.014	0.032	1.9
7.45	[M+H]+	496.3407	496.3403	0.0004	LPC(16:0)	0.19220	0.20277	0.18155	0.10363	0.11453	0.10569	0.06916	0.67675	0.11160	0.14735	0.988	0.988	-
5.92	[M+H]+	494.3248	494.3241	0.0007	LPC(16:1)	0.02108	0.03124	0.02951	0.01686	0.01423	0.01730	0.01970	0.17548	0.02226	0.02827	0.385	0.493	-
8.92	[M+H]+	524.3723	524.3716	0.0007	LPC(18:0)	0.02687	0.02697	0.02832	0.01342	0.01633	0.01723	0.00787	0.07054	0.01157	0.01482	0.631	0.711	-
7.76	[M+H]+	522.3558	522.3559	0.0001	LPC(18:1)	0.10235	0.07614	0.12546	0.03804	0.04231	0.03356	0.05344	0.53650	0.08115	0.01906	0.986	0.988	-
9.07	[M+H]+	550.3875	550.3867	0.0008	LPC(20:1)	0.00557	0.00623	0.00688	0.00336	0.00367	0.00496	0.00372	0.03598	0.00556	0.00762	0.319	0.432	-
14.19	[M+H]+	706.5350	706.5387	0.0037	PC(14:0-16:0)	2.16198	2.17916	2.48730	1.28463	1.31154	1.15146	2.01159	1.87080	1.17333	1.74368	0.408	0.506	-
15.76	[M+H]+	734.5629	734.5700	0.0071	PC(16:0/16:0)	0.80140	0.05493	0.25559	0.13336	0.47603	0.03637	0.84165	0.03596	0.48875	0.05140	0.456	0.551	-
15.75	[M+H]+	760.5779	760.5856	0.0077	PC(16:0-18:1)	5.46140	5.46184	5.78045	3.24104	3.28987	3.34168	5.61236	5.28933	3.29846	4.86705	0.873	0.910	-
13.39	[M+H]+	730.5374	730.5387	0.0013	PC(16:1/16:1)	2.52199	2.53006	2.91783	1.40021	1.48999	3.07804	5.30833	5.15300	3.14254	4.70143	0.007	0.017	1.0
14.69	[M+H]+	758.5637	758.5700	0.0063	PC(16:1-18:1)	3.43603	3.44394	3.88446	2.07404	2.08145	3.52753	5.87264	5.59140	3.50381	5.23421	0.028	0.054	0.7
17.45	[M+H]+	788.6092	788.6169	0.0077	PC(18:0-18:1)	1.13062	1.14763	1.23598	0.61104	0.66149	0.71917	1.09575	1.01060	0.62620	0.83054	0.638	0.713	-
15.94	[M+H]+	786.5926	786.6013	0.0087	PC(18:1/18:1)	2.80195	2.79919	3.12470	1.68080	1.68865	2.42438	4.08792	3.90297	2.41918	3.61418	0.113	0.184	-

Continued

RT (min)	Adduct ion	row m/z	theo m/z	delta m/z	Lipids	Adherent_1	Adherent_2	Adherent_3	Adherent_4	Adherent_5	Sphere_1	Sphere_2	Sphere_3	Sphere_4	Sphere_5	p.value	FDR	log2(Fc)

14.90	[M+H]+	784.5795	784.5856	0.0061	PC(18:1-18:2)	0.36488	0.37788	0.29732	0.10491	0.22143	0.40334	0.66750	0.64102	0.39790	0.58482	0.021	0.043	1.0
13.01	[M+H]+	678.5082	678.5074	0.0008	PC(28:0)	1.12303	1.11086	1.28900	0.60543	0.66454	0.37878	0.66530	0.63385	0.38835	0.56964	0.018	0.037	-0.9
12.07	[M+H]+	676.4923	676.4917	0.0006	PC(28:1)	0.02278	0.02261	0.02416	0.01239	0.01331	0.01607	0.02862	0.02680	0.01743	0.02520	0.331	0.438	-
13.50	[M+H]+	692.5227	692.5230	0.0003	PC(29:0)	0.28121	0.28591	0.24798	0.15025	0.16512	0.04464	0.07605	0.07439	0.04324	0.06538	0.000	0.000	-1.9
13.09	[M+H]+	704.5225	704.5230	0.0005	PC(30:1)	3.07582	3.10765	3.59314	1.71382	1.83216	2.03719	1.70626	1.65806	0.95864	3.14094	0.172	0.262	-
12.33	[M+H]+	702.5068	702.5074	0.0006	PC(30:2)	0.01296	0.00826	0.01394	0.00461	0.00503	0.01377	0.02317	0.02314	0.01362	0.00761	0.080	0.134	-
14.50	[M+H]+	720.5482	720.5543	0.0061	PC(31:0)	0.07173	0.07094	0.08246	0.03931	0.04040	0.01333	0.02364	0.02146	0.01355	0.02070	0.000	0.001	-1.7
13.86	[M+H]+	718.5381	718.5387	0.0006	PC(31:1)	0.74423	0.74775	0.85697	0.40000	0.44169	0.18474	0.31441	0.31012	0.18032	0.27129	0.002	0.005	-1.3
14.42	[M+H]+	732.5495	732.5543	0.0048	PC(32:1)	6.63621	6.58136	4.37350	3.96673	3.97177	3.97459	6.89162	6.47826	4.08633	6.10506	0.657	0.721	-
16.28	[M+H]+	748.5767	748.5856	0.0089	PC(33:0)	0.12553	0.13291	0.14833	0.07419	0.07696	0.02570	0.04401	0.04164	0.02542	0.03844	0.000	0.001	-1.7
13.71	[M+H]+	756.5512	756.5543	0.0031	PC(34:3)	0.31215	0.31235	0.35567	0.17228	0.18433	0.41337	0.22502	0.68097	0.41422	0.61000	0.061	0.107	-
13.12	[M+H]+	754.5351	754.5387	0.0036	PC(34:4)	0.17122	0.16812	0.17926	0.00580	0.10284	0.06301	0.10947	0.10386	0.06656	0.09600	0.909	0.939	-
16.54	[M+H]+	774.5921	774.6013	0.0092	PC(35:1)	0.44207	0.01561	0.42758	0.24771	0.26100	0.11790	0.19884	0.00216	0.11540	0.16807	0.346	0.448	-
14.38	[M+H]+	782.5633	782.5700	0.0067	PC(36:4)	0.15409	0.18483	0.30790	0.10926	0.17849	0.05300	0.07499	0.07395	0.04720	0.06816	0.001	0.002	-1.6
13.48	[M+H]+	780.5510	780.5543	0.0033	PC(36:5)	0.22301	0.22138	0.23340	0.11779	0.13212	0.01235	0.02903	0.01329	0.00752	0.02524	0.000	0.000	-3.4
17.90	[M+H]+	802.6247	802.6326	0.0079	PC(37:1)	0.03246	0.00287	0.02888	0.01747	0.01939	0.01257	0.02087	0.02014	0.01187	0.01704	0.946	0.962	-
16.86	[M+H]+	800.6184	800.6169	0.0015	PC(37:2)	0.00884	0.00816	0.00839	0.00442	0.00478	0.00220	0.00338	0.00339	0.00198	0.00282	0.001	0.004	-1.3
17.44	[M+H]+	814.6244	814.6326	0.0082	PC(38:2)	0.48481	0.49892	0.55030	0.26667	0.28083	0.42130	0.72585	0.68260	0.41120	0.58720	0.135	0.213	-
17.26	[M+H]+	812.6188	812.6169	0.0019	PC(38:3)	0.00056	0.00008	0.00029	0.00112	0.00165	0.00024	0.00033	0.00055	0.00035	0.00038	0.643	0.713	-
15.26	[M+H]+	810.5932	810.6013	0.0081	PC(38:4)	0.07182	0.07407	0.08435	0.03965	0.04294	0.01549	0.02945	0.02134	0.01051	0.02490	0.001	0.004	-1.6

Continued

RT (min)	Adduct ion	row m/z	theo m/z	delta m/z	Lipids	Adherent_1	Adherent_2	Adherent_3	Adherent_4	Adherent_5	Sphere_1	Sphere_2	Sphere_3	Sphere_4	Sphere_5	p.value	FDR	log2(Fc)

14.51	[M+H]+	808.5786	808.5856	0.0070	PC(38:5)	0.27334	0.27605	0.14845	0.16256	0.17030	0.03184	0.03491	0.03318	0.02162	0.00482	0.000	0.002	-3.0
14.27	[M+H]+	806.5653	806.5700	0.0047	PC(38:6)	0.27442	0.28386	0.07520	0.16141	0.17299	0.04481	0.02427	0.02064	0.01416	0.01943	0.000	0.001	-3.0
12.93	[M+H]+	804.5537	804.5543	0.0006	PC(38:7)	0.05255	0.05141	0.05240	0.02748	0.03112	0.00075	0.00088	0.00084	0.00069	0.00089	0.000	0.000	-5.7
18.73	[M+H]+	842.6583	842.6639	0.0056	PC(40:2)	0.05588	0.05433	0.06286	0.02902	0.03206	0.03076	0.05786	0.04698	0.01701	0.04850	0.505	0.592	-
15.00	[M+H]+	834.5932	834.6007	0.0075	PC(40:6)	0.00817	0.06760	0.02489	0.03876	0.04180	0.00796	0.00603	0.00568	0.00358	0.00494	0.002	0.006	-2.7
14.16	[M+H]+	832.5781	832.5856	0.0075	PC(40:7)	0.19792	0.20078	0.21840	0.11735	0.12565	0.00442	0.00615	0.00600	0.00404	0.00546	0.000	0.000	-5.0
13.26	[M+H]+	830.5673	830.5700	0.0027	PC(40:8)	0.00833	0.00816	0.00853	0.00432	0.00504	0.00028	0.00025	0.00023	0.00016	0.00024	0.000	0.000	-4.9
20.15	[M+H]+	870.6890	870.6952	0.0062	PC(42:2)	0.04958	0.02368	0.05744	0.00557	0.01223	0.02040	0.03646	0.03566	0.02060	0.03100	0.583	0.670	-
14.10	[M+H]+	858.5955	858.6013	0.0058	PC(42:8)	0.00153	0.00166	0.00186	0.00098	0.00105	0.00004	0.00006	0.00004	0.00004	0.00004	0.000	0.000	-5.1
13.87	[M+H]+	664.5246	664.5281	0.0035	PC(O-28:0)	0.00294	0.00269	0.00296	0.00155	0.00165	0.00023	0.00018	0.00029	0.00018	0.00020	0.000	0.000	-3.4
14.32	[M+H]+	678.5391	678.5437	0.0046	PC(O-29:0)	0.00011	0.00005	0.00006	0.00008	0.00005	0.00001	0.00001	0.00001	0.00000	0.00001	0.000	0.000	-3.4
16.80	[M+H]+	720.5817	720.5907	0.0090	PC(O-32:0)	0.00368	0.00405	0.00417	0.00198	0.00187	0.00001	0.00001	0.00000	0.00000	0.00000	0.000	0.000	-9.2
18.96	[M+H]+	748.6139	748.6220	0.0081	PC(O-34:0)	0.00001	0.00004	0.00007	0.00004	0.00003	0.00000	0.00002	0.00000	0.00000	0.00000	0.002	0.006	-2.9
14.99	[M+H]+	818.5976	818.6063	0.0087	PC(O-40:7)	0.00001	0.00013	0.00001	0.00001	0.00000	0.00004	0.00007	0.00006	0.00001	0.00007	0.179	0.266	-
16.13	[M-H]-	716.5264	716.5236	0.0028	PE(16:0-18:1) + PE(16:1-18:0)	13.21690	14.10126	13.44505	16.44676	16.23264	13.41940	10.69481	10.58532	13.49184	11.13568	0.017	0.037	-0.3
14.72	[M-H]-	738.5118	738.5079	0.0039	PE(16:0-20:4)	3.09952	3.24912	3.22666	3.92056	4.88500	0.60325	0.54020	0.49326	0.64993	0.51478	0.000	0.000	-2.7
13.61	[M-H]-	686.4800	686.4766	0.0034	PE(16:1/16:1)	1.02441	1.08415	1.08109	1.31304	1.29848	3.98628	3.23333	3.22992	4.11097	3.58447	0.000	0.000	1.6
17.68	[M-H]-	744.5607	744.5549	0.0058	PE(18:0-18:1)	7.98581	8.55515	8.17684	10.23152	10.03026	5.86195	4.40386	4.75740	5.74619	4.95021	0.000	0.000	-0.8
16.72	[M-H]-	768.5532	768.5549	0.0017	PE(18:0-20:3a) + PE(18:1-20:2)	0.08436	0.11130	1.05899	1.31300	1.33355	0.81477	0.62209	0.64817	0.82844	0.68698	0.471	0.557	-

Continued

RT (min)	Adduct ion	row m/z	theo m/z	delta m/z	Lipids	Adherent_1	Adherent_2	Adherent_3	Adherent_4	Adherent_5	Sphere_1	Sphere_2	Sphere_3	Sphere_4	Sphere_5	p.value	FDR	log2(Fc)
17.34	[M-H]-	768.5532	768.5549	0.0017	PE(18:0-20:3b)	0.06930	0.07698	0.07018	0.09708	0.09792	0.04389	0.03297	0.03105	0.06088	0.03023	0.001	0.004	-1.0
16.23	[M-H]-	742.5436	742.5392	0.0044	PE(18:1/18:1)	31.72315	33.26521	31.88965	39.58231	38.75855	43.99649	34.22331	34.20096	43.73264	35.94998	0.265	0.366	-
15.27	[M-H]-	740.5261	740.5236	0.0025	PE(18:1-18:2)	1.92500	2.04723	1.94159	2.48635	2.38994	4.02262	3.05577	3.19018	4.07979	3.33188	0.000	0.001	0.7
17.60	[M-H]-	770.5724	770.5705	0.0019	PE(18:1-20:1)	1.21455	1.24884	1.23018	1.56091	1.50480	1.66392	1.21500	1.35181	1.61687	1.41359	0.398	0.500	-
15.79	[M-H]-	698.5130	698.5130	0.0000	PE(O-16:1/18:2) + PE(O-18:2/16:1)	0.29855	0.32173	0.30084	0.35761	0.35229	0.00274	0.00103	0.00179	0.00210	0.00106	0.000	0.000	-7.5
15.42	[M-H]-	722.5090	722.5130	0.0040	PE(O-16:1/20:4)	0.29389	0.32051	0.32356	0.36104	0.36326	0.00123	0.00058	0.00000	0.00000	0.00000	0.000	0.001	-9.8
14.47	[M-H]-	720.4927	720.4974	0.0047	PE(O-16:1/20:5) + PE(O-16:2/20:4)	0.08567	0.09986	0.09263	0.11287	0.11259	0.00032	0.00045	0.00111	0.00105	0.00056	0.000	0.000	-7.2
17.41	[M-H]-	752.5647	752.5600	0.0047	PE(O-18:0/20:4) + PE(O-18:1/20:3)	0.01001	0.00893	0.00967	0.01282	0.01069	0.00000	0.00000	0.00000	0.00000	0.00000	0.000	0.000	-13.5
18.42	[M-H]-	702.5471	702.5443	0.0028	PE(O-18:1/16:0) + PE(O-16:1/18:0)	0.00342	0.00562	0.00666	0.00745	0.00782	0.00000	0.00000	0.00000	0.00000	0.00000	0.000	0.000	-12.8
18.36	[M-H]-	728.5645	728.5600	0.0045	PE(O-18:1/18:1)	0.17620	0.18249	0.17638	0.22111	0.21323	0.00000	0.00025	0.00000	0.00000	0.00000	0.000	0.000	-11.9
16.77	[M-H]-	750.5482	750.5443	0.0039	PE(O-18:1/20:4)	0.12248	0.13257	0.12698	0.15440	0.15093	0.01015	0.00906	0.00695	0.00922	0.00684	0.000	0.000	-4.0
17.05	[M-H]-	726.5482	726.5443	0.0039	PE(O-18:2/18:1)	0.15094	0.16083	0.15054	0.19008	0.18312	0.00000	0.00000	0.00000	0.00000	0.00000	0.000	0.000	-17.5
12.88	[M+H]+	675.5430	675.5422	0.0008	SM(d32:1)	0.05695	0.05742	0.06327	0.03211	0.03617	0.04267	0.07155	0.06822	0.04268	0.05997	0.395	0.500	-
14.90	[M+H]+	705.5843	705.5910	0.0067	SM(d34:0)	0.14815	0.13190	0.16216	0.08763	0.08717	0.08480	0.13759	0.12899	0.08128	0.11471	0.539	0.625	-
14.23	[M+H]+	703.5706	703.5754	0.0048	SM(d34:1)	1.52472	0.00729	0.06812	0.95107	0.00371	1.32378	2.30268	2.22549	1.39656	1.87768	0.034	0.062	1.8
13.08	[M+H]+	701.5537	701.5597	0.0060	SM(d34:2)	0.10222	0.10146	0.01772	0.05806	0.06443	0.02782	0.04372	0.04581	0.02695	0.03747	0.178	0.266	-
15.66	[M+H]+	731.6017	731.6067	0.0050	SM(d36:1)	0.00071	0.00053	0.00712	0.00275	0.00055	0.00049	0.00052	0.00057	0.00031	0.00045	0.080	0.134	-
18.89	[M+H]+	787.6612	787.6693	0.0081	SM(d40:1)	0.13361	0.13711	0.14789	0.06826	0.07705	0.05424	0.09095	0.08907	0.05460	0.04090	0.044	0.080	-0.8
20.29	[M+H]+	815.6939	815.7006	0.0067	SM(d42:1)	0.31877	0.32933	0.35327	0.16048	0.18182	0.07635	0.13333	0.13067	0.07738	0.09830	0.002	0.005	-1.4

Continued

RT (min)	Adduct ion	row m/z	theo m/z	delta m/z	Lipids	Adherent_1	Adherent_2	Adherent_3	Adherent_4	Adherent_5	Sphere_1	Sphere_2	Sphere_3	Sphere_4	Sphere_5	p.value	FDR	log2(Fc)
18.69	[M+H]+	813.6772	813.6849	0.0077	SM(d42:2a)	1.04417	0.44401	1.17771	0.54382	0.60630	0.31388	1.47169	1.43009	0.86734	1.20765	0.457	0.551	-
19.78	[M+H]+	813.6790	813.6849	0.0059	SM(d42:2b)	0.01481	0.00568	0.01584	0.00748	0.00310	0.00937	0.01556	0.01520	0.00918	0.01261	0.230	0.330	-
21.81	[M+H]+	843.7227	843.7319	0.0092	SM(d44:1)	0.04404	0.04516	0.04557	0.01317	0.00931	0.00656	0.01101	0.01076	0.00662	0.00930	0.018	0.037	-1.8
20.16	[M+H]+	841.7089	841.7162	0.0073	SM(d44:2)	0.34294	0.34246	0.13121	0.16901	0.19248	0.10557	0.17726	0.17888	0.10525	0.10778	0.056	0.100	-
24.44	[M+NH4]+	796.7320	796.7394	0.0074	TG(14:0-16:0-16:0)	0.00273	0.00210	0.00195	0.00100	0.00112	0.00099	0.00071	0.00149	0.00125	0.00156	0.168	0.258	-
25.79	[M+NH4]+	852.7945	852.8020	0.0075	TG(16:0-16:0-18:0)	0.00140	0.00107	0.00094	0.00048	0.00049	0.00036	0.00024	0.00053	0.00041	0.00053	0.029	0.056	-1.1
26.63	[M+NH4]+	880.8262	880.8333	0.0071	TG(16:0-18:0-18:0)	0.00043	0.00033	0.00030	0.00016	0.00018	0.00013	0.00008	0.00021	0.00015	0.00023	0.070	0.121	-
24.86	[M+NH4]+	876.7915	876.8015	0.0100	TG(16:0-18:1-18:1)	0.11394	0.08614	0.08509	0.04249	0.04726	0.05157	0.03792	0.08039	0.06480	0.06850	0.469	0.557	-
22.89	[M+NH4]+	818.7166	818.7238	0.0072	TG(16:1/16:1/16:1) +TG(14:1-16:1-18:1)	0.00201	0.00156	0.00141	0.00071	0.00079	0.00737	0.00572	0.01168	0.00952	0.01037	0.000	0.000	2.8
23.59	[M+NH4]+	846.7481	846.7551	0.0070	TG(16:1-16:1-18:1)	0.02047	0.01579	0.01566	0.00734	0.00831	0.02912	0.02216	0.04599	0.03765	0.04084	0.003	0.008	1.4
24.22	[M+NH4]+	874.7792	874.7864	0.0072	TG(16:1-18:1-18:1)	0.06755	0.05120	0.05100	0.02568	0.02818	0.04983	0.03760	0.07949	0.06399	0.06849	0.192	0.278	-
24.80	[M+NH4]+	902.8091	902.8177	0.0086	TG(18:1/18:1/18:1)	0.08735	0.06767	0.06617	0.03336	0.03631	0.04284	0.03224	0.06838	0.05357	0.05858	0.694	0.755	-
24.21	[M+NH4]+	900.7945	900.8015	0.0070	TG(18:1-18:1-18:2)	0.00867	0.00630	0.00642	0.00242	0.00231	0.00594	0.00447	0.00936	0.00671	0.00818	0.235	0.332	-
23.83	[M+NH4]+	950.8079	950.8177	0.0098	TG(18:1-18:1-22:5)	0.00522	0.00409	0.00398	0.00180	0.00214	0.00008	0.00007	0.00013	0.00012	0.00012	0.000	0.000	-5.0
23.55	[M+NH4]+	948.7928	948.8020	0.0092	TG(18:1-18:1-22:6)	0.00396	0.00306	0.00305	0.00139	0.00163	0.00004	0.00002	0.00007	0.00004	0.00005	0.000	0.000	-5.8
23.76	[M+NH4]+	768.7026	768.7081	0.0055	TG(44:0)	0.00096	0.00070	0.00070	0.00031	0.00040	0.00037	0.00028	0.00058	0.00049	0.00055	0.329	0.438	-
23.67	[M+NH4]+	794.7172	794.7238	0.0066	TG(46:1)	0.00745	0.00581	0.00548	0.00253	0.00298	0.00423	0.00316	0.00642	0.00543	0.00576	0.747	0.792	-
22.91	[M+NH4]+	792.7018	792.7081	0.0063	TG(46:2)	0.00201	0.00160	0.00156	0.00050	0.00080	0.00427	0.00325	0.00647	0.00547	0.00577	0.001	0.003	2.0
24.02	[M+NH4]+	808.7324	808.7389	0.0065	TG(47:1)	0.00193	0.00155	0.00145	0.00069	0.00080	0.00043	0.00031	0.00063	0.00055	0.00071	0.009	0.020	-1.3

Continued

RT (min)	Adduct ion	row m/z	theo m/z	delta m/z	Lipids	Adherent_1	Adherent_2	Adherent_3	Adherent_4	Adherent_5	Sphere_1	Sphere_2	Sphere_3	Sphere_4	Sphere_5	p.value	FDR	log2(Fc)
24.35	[M+NH4]+	822.7481	822.7551	0.0070	TG(48:1)	0.02432	0.01804	0.01745	0.00869	0.00971	0.01040	0.00778	0.01575	0.01301	0.01420	0.412	0.507	-
23.64	[M+NH4]+	820.7331	820.7394	0.0063	TG(48:2)	0.02251	0.01732	0.01704	0.00806	0.00919	0.01927	0.01472	0.02993	0.02492	0.02661	0.073	0.124	-
24.64	[M+NH4]+	836.7628	836.7707	0.0079	TG(49:1)	0.00403	0.00165	0.00294	0.00140	0.00161	0.00071	0.00052	0.00108	0.00088	0.00109	0.005	0.012	-1.4
23.96	[M+NH4]+	834.7477	834.7551	0.0074	TG(49:2)	0.00312	0.00252	0.00237	0.00107	0.00128	0.00067	0.00030	0.00103	0.00043	0.00101	0.008	0.018	-1.6
24.99	[M+NH4]+	850.7788	850.7864	0.0076	TG(50:1)	0.03185	0.02456	0.02312	0.01148	0.01289	0.01215	0.00895	0.01883	0.01497	0.01627	0.191	0.278	-
24.26	[M+NH4]+	848.7610	848.7707	0.0097	TG(50:2)	0.07637	0.05939	0.05706	0.02983	0.03227	0.04206	0.03213	0.06474	0.05345	0.05815	0.939	0.962	-
23.93	[M+NH4]+	860.7627	860.7707	0.0080	TG(51:3)	0.00202	0.00040	0.00155	0.00068	0.00083	0.00047	0.00034	0.00074	0.00056	0.00067	0.124	0.199	-
25.65	[M+NH4]+	878.8099	878.8177	0.0078	TG(52:1)	0.01259	0.00989	0.00881	0.00059	0.00475	0.00289	0.00207	0.00436	0.00337	0.00088	0.269	0.368	-
23.61	[M+NH4]+	872.7636	872.7707	0.0071	TG(52:4)	0.00395	0.00310	0.00304	0.00143	0.00162	0.00485	0.00367	0.00756	0.00624	0.00666	0.008	0.018	1.1
25.96	[M+NH4]+	892.8244	892.8333	0.0089	TG(53:1)	0.00088	0.00074	0.00068	0.00031	0.00038	0.00013	0.00010	0.00022	0.00016	0.00021	0.001	0.003	-1.9
24.52	[M+NH4]+	888.7934	888.8020	0.0086	TG(53:3)	0.00501	0.00395	0.00367	0.00171	0.00198	0.00078	0.00058	0.00106	0.00093	0.00097	0.001	0.002	-1.9
23.97	[M+NH4]+	886.7779	886.7864	0.0085	TG(53:4)	0.00059	0.00047	0.00045	0.00022	0.00026	0.00013	0.00008	0.00019	0.00015	0.00018	0.004	0.010	-1.4
26.41	[M+NH4]+	906.8408	906.8490	0.0082	TG(54:1)	0.00184	0.00140	0.00132	0.00064	0.00071	0.00046	0.00033	0.00072	0.00060	0.00063	0.019	0.040	-1.1
25.53	[M+NH4]+	904.8257	904.8333	0.0076	TG(54:2)	0.03542	0.02782	0.02541	0.01190	0.01347	0.00981	0.00720	0.01502	0.01165	0.01262	0.030	0.057	-1.0
23.72	[M+NH4]+	898.7780	898.7864	0.0084	TG(54:5)	0.00224	0.00178	0.00184	0.00083	0.00095	0.00096	0.00074	0.00148	0.00123	0.00138	0.344	0.448	-
23.32	[M+NH4]+	896.7615	896.7707	0.0092	TG(54:6)	0.00248	0.00194	0.00188	0.00086	0.00105	0.00011	0.00009	0.00016	0.00014	0.00017	0.000	0.000	-3.6
22.86	[M+NH4]+	894.7477	894.7551	0.0074	TG(54:7)	0.00142	0.00109	0.00088	0.00049	0.00060	0.00005	0.00004	0.00008	0.00005	0.00007	0.000	0.000	-3.9
23.60	[M+NH4]+	910.7780	910.7864	0.0084	TG(55:6)	0.00047	0.00037	0.00036	0.00017	0.00020	0.00001	0.00001	0.00002	0.00002	0.00002	0.000	0.000	-4.4
25.40	[M+NH4]+	930.8405	930.8490	0.0085	TG(56:3)	0.01601	0.01693	0.01607	0.00746	0.00840	0.00980	0.00745	0.01554	0.01175	0.01347	0.714	0.770	-

Continued

RT (min)	Adduct ion	row m/z	theo m/z	delta m/z	Lipids	Adherent_1	Adherent_2	Adherent_3	Adherent_4	Adherent_5	Sphere_1	Sphere_2	Sphere_3	Sphere_4	Sphere_5	p.value	FDR	log2(Fc)
24.78	[M+NH4]+	928.8242	928.8333	0.0091	TG(56:4)	0.00115	0.00205	0.00095	0.00042	0.00077	0.00105	0.00091	0.00234	0.00184	0.00130	0.238	0.332	-
24.45	[M+NH4]+	926.8090	926.8177	0.0087	TG(56:5)	0.00067	0.00088	0.00079	0.00050	0.00025	0.00008	0.00004	0.00004	0.00002	0.00004	0.000	0.000	-3.8
23.87	[M+NH4]+	924.7947	924.8020	0.0073	TG(56:6)	0.00567	0.00449	0.00427	0.00186	0.00227	0.00013	0.00010	0.00020	0.00014	0.00018	0.000	0.000	-4.6
23.44	[M+NH4]+	922.7785	922.7864	0.0079	TG(56:7)	0.00190	0.00128	0.00151	0.00065	0.00081	0.00004	0.00003	0.00006	0.00006	0.00007	0.000	0.000	-4.6
22.89	[M+NH4]+	920.7627	920.7702	0.0075	TG(56:8)	0.00111	0.00089	0.00089	0.00037	0.00046	0.00004	0.00002	0.00005	0.00004	0.00004	0.000	0.000	-4.3
25.69	[M+NH4]+	944.8554	944.8646	0.0092	TG(57:3)	0.00097	0.00080	0.00052	0.00032	0.00037	0.00024	0.00017	0.00037	0.00026	0.00034	0.021	0.043	-1.1
27.07	[M+NH4]+	960.8876	960.8954	0.0078	TG(58:2)	0.00429	0.00326	0.00325	0.00147	0.00172	0.00159	0.00113	0.00247	0.00185	0.00215	0.166	0.258	-
26.09	[M+NH4]+	958.8723	958.8797	0.0074	TG(58:3)	0.00584	0.00672	0.00319	0.00189	0.00355	0.00237	0.00196	0.00384	0.00386	0.00701	0.737	0.788	-
25.38	[M+NH4]+	956.8549	956.8646	0.0097	TG(58:4)	0.00211	0.00162	0.00159	0.00073	0.00083	0.00101	0.00077	0.00130	0.00125	0.00141	0.623	0.709	-
24.94	[M+NH4]+	954.8396	954.8484	0.0088	TG(58:5)	0.00034	0.00016	0.00021	0.00005	0.00010	0.00001	0.00003	0.00008	0.00004	0.00007	0.020	0.040	-1.9

Supplementary Table 3. Features with VIP scores of 1 or higher.

**Compound**	**VIP score**
PEO-18:2/18:1	2.7079
PEO-18:1/18:1	2.5836
PEO-16:1/20:4	2.4774
PEO-18:0/20:4 or PEO-18:1/20:3	2.2061
PEO-16:1/18:2 or PEO-18:2/16:1	2.0295
PEO-18:1/16:0 or PEO-16:1/18:0	2.0063
PEO-16:1/20:5 or PEO-16:2/20:4	1.858
PCO-40:7	1.6907
PCO-32:0	1.6645
TG16:1/16:1/16:1 or TG14:1-16:1-18:1	1.6344
PC38:7	1.576
PC40:7	1.5621
Cerd18:1/16:0	1.5064
TG46:2	1.4789
SMd34:1	1.4429
PCO-34:0	1.3865
PE16:0-20:4	1.3607
PEO-18:1/20:4	1.3074
PC40:8	1.2865
Cerd18:1/14:0	1.2788
TG16:1-16:1-18:1	1.2752
TG52:4	1.2535
TG16:0-18:0-18:0	1.2346
TG56:4	1.2043
PC36:5	1.1816
Cerd18:1/24:1	1.1571
TG18:1-18:1-22:6	1.1522
TG58:5	1.1468
TG18:1-18:1-22:5	1.1226
PCO-29:0	1.1206
TG44:0	1.1157
TG16:1-18:1-18:1	1.0977
PC3:02	1.0806
TG56:6	1.0746
TG48:2	1.0578
PE16:1/16:1	1.0553
TG53:4	1.0529
TG58:4	1.0286
TG18:1-18:1-18:2	1.0019

Supplementary Table 4. Altered genes involved in unsaturated fatty acid biosynthesis between adherent and sphere MCF-7 cells.

**Genes**	**Fold change**
PTPLB	-1.22
PECR	-0.96
ELOVL2	-0.63
ACOT4	-0.46
ACOX1	-0.24
ACOT7	-0.22
ELOVL6	0.2
HADHA	0.47
FADS1	0.8
ACOX3	0.84
SCD	1.88
